# Isomalabaricane Chemical Composition of Vietnamese Marine Sponges Inspected by Metabolomic and Chemical Approaches

**DOI:** 10.3390/md23120466

**Published:** 2025-12-05

**Authors:** Sophia A. Kolesnikova, Anastasia B. Kozhushnaya, Vladimir A. Shilov, Andrey D. Kukhlevsky, Anatoly I. Kalinovsky, Roman S. Popov, Pavel S. Dmitrenok, Natalia V. Ivanchina

**Affiliations:** 1 G.B. Elyakov Pacific Institute of Bioorganic Chemistry, Far Eastern Branch, Russian Academy of Sciences, Pr. 100-Let Vladivostoku 159, 690022 Vladivostok, Russia; kozhushnaia.ab@mail.ru (A.B.K.); kaaniw@piboc.dvo.ru (A.I.K.); popov_rs@piboc.dvo.ru (R.S.P.); ivanchina@piboc.dvo.ru (N.V.I.); 2 A.V. Zhirmunsky National Scientific Center of Marine Biology, Far Eastern Branch, Russian Academy of Sciences, ul. Palchevskogo 17, 690041 Vladivostok, Russia; shilvl@yandex.ru (V.A.S.); ad.kukhlevskiy@gmail.com (A.D.K.)

**Keywords:** *Rhabdastrella globostellata*, marine sponges, isomalabaricanes, triterpenoids, chemotaxonomy, 18S rRNA, 28S rRNA, LC–MS, molecular networking, metabolomics

## Abstract

Reliable taxonomy of biological producers is essential for finding new natural substances. A recent study morphologically re-examined 21 accessed vouchers to confirm multiple reported misidentifications and suggested marine sponges from the genus *Rhabdastrella* as the only known source of the isomalabaricane triterpenoids. The present study aimed to find isomalabaricane-containing sponges among the samples collected during seven marine expeditions to the Vietnam waters of the South China Sea, accompanied with their identification confirmed using morphological and molecular (18S rRNA and 28S rRNA) analyses. As a result, nine sponges identified as *Rhabdastrella globostellata* were found to contain isomalabaricanes in their extracts. A chemical investigation of the *R. globostellata* (PIBOC O63-136) specimen led to the isolation of nine isomalabaricane triterpenoids including the new compound **1**, of which the chemical structure was elucidated based on HRESIMS and NMR data. Subsequently, a combination of LC–MS/MS, multivariate statistical analysis, and feature-based molecular networking was applied to detect, annotate, and characterize the isomalabaricane chemical diversity across the nine *R. globostellata* specimens. As a result, two primary chemotypes containing individual sets of annotated compounds were discovered within the Vietnamese population of this sponge. Moreover, obtained data showed a series of new extremely rare isomalabaricanes in *R. globostellata* extracts including nitrogen-containing metabolites and glycosides of this structural class.

## 1. Introduction

Isomalabaricanes and their *nor*-derivatives constitute an ever-growing group of marine natural triterpenoids, which currently includes more than 200 metabolites. In general, isomalabaricanes have a C_30_ skeleton constructed of a *trans*-*syn*-*trans* 6,6,5-tricyclic core connected with a polyunsaturated or oxidized side chain. In most of them, a characteristic feature is an oxidized core with an oxygen-containing function at the C-3 position and a 12-keto group [1,2,3]. Less common *nor*-isomalabaricanes have shortened side chains or even lose the 5-membered C cycle due to further oxidation [4,5].

Some compounds of the isomalabaricane class have been shown as promising pharmacological agents that exert effects at extremely low concentrations. The best illustration is two isomeric stellettins A and B that are known for their significant and selective activity at nanomolar concentrations against murine leukemia P388 and human glioblastoma SF295 cells, respectively [6,7]. A recent study has provided evidence that stellettin B suppresses the viability of bladder cancer cells via activation of the autophagy/DAPK2/apoptosis signaling cascade without severe toxicity towards normal uroepithelial cells [8]. On the other hand, nanomolar concentration of this triterpenoid has demonstrated neuroprotective effects in vitro and in vivo using 6-OHDA treated SH-SY5Y cells and the zebrafish model of Parkinson’s disease [9]. These findings stimulate researchers’ interest in structural and pharmacological studies of isomalabaricanes, as well as the search for new biological sources of these metabolites.

Since the first isolation in 1981 [10], all terpenoids of this type have been reported from tropical marine sponges belonging to four genera—*Geodia*, *Jaspis*, *Rhabdastrella*, and *Stelletta* [1,2,3]. However, a recent re-examination of 21 sponge vouchers accessed from these publications concluded that isomalabaricanes had been isolated from only two *Rhabdastrella* species and, possibly, one *Geodia* species [11]. The Vietnamese sponge PIBOC O38-301, which we reported as a source of diverse active isomalabaricane metabolites [5,12,13,14], was also re-identified by Dr. Paco Cárdenas as *R. globostellata*.

We believe that reliable taxonomy of biological organisms is essential for assessing the potential of the source and understanding the biosynthetic pathways and ecological functions of target secondary metabolites. Our present study aimed to search for isomalabaricane-containing sponges among the specimens collected during the expeditions to Vietnam waters of the South China Sea on board the R/V “Akademik Oparin” with their identification confirmed using morphological and genetic (18S rRNA and 28S rRNA) analyses. Nine isomalabaricane triterpenoids **1**–**9** were isolated from the *R. globostellata* (PIBOC O63-136), including one new compound **1**. Its chemical structure was elucidated based on the HRESIMS and NMR data. Since a series of individual isomalabaricanes was available to us, we used the compounds for LC–ESI MS and molecular networking for characterizing the sponge extracts.

## 2. Results

### 2.1. Chemical Screening and Systematic Identification of Sponge Vouchers

**Figure 1:**
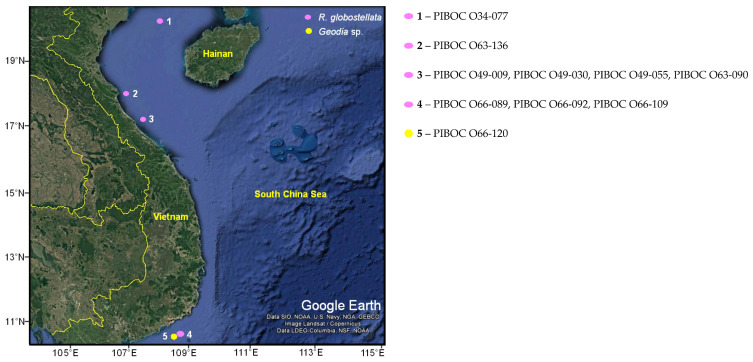
Map of the study area and localities of sponge samples.

To search for sponges containing isomalabaricanes, we screened the samples collected during seven marine expeditions in 2007–2023. Along with the photographs and biological description of the specimens, thin-layer chromatograms (TLC) of their EtOH extracts were obtained during those expeditions. We used these data to select sponges that visually related to the genera *Geodia*, *Jaspis*, *Rhabdastrella*, or *Stelletta* and had a bright yellow pigment in the extract characteristic of some isomalabaricanes [1,10]. At first glance, of the ten Vietnamese sponges found (Table 1, Figure 1), nine were assigned to the genus *Rhabdastrella* (Figure S1) and one to the genus *Geodia* (Figure S2).

**Figure 2:**
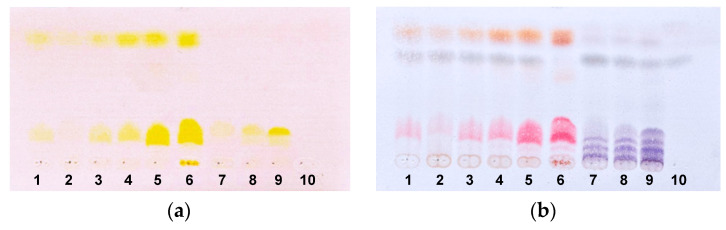
TLCs on silica gel plates of ten EtOH sponge extracts (CHCl_3_/EtOH, 20:1) (**a**) before and (**b**) after treatment with H_2_SO_4_ aerosol and heating.

Fresh-made TLCs in CHCl_3_/EtOH (20:1) confirmed the bright yellow pigments in all *Rhabdastrella* samples and showed two groups of these sponges in accordance with similarity in chemical components of their EtOH extracts (Figure 2). The larger group included six specimens 1–6 similar to the previously studied sponge *R. globostellata* (PIBOC O38-301, Figure S3). The *Geodia* extract 10 was significantly different, because no pigments were detected.

Below is a systematic description based on morphological analysis of nine *Rhabdastrella* specimens:

Phylum **Porifera** Grant, 1836;

Class **Demospongiae** Sollas, 1885;

Subclass **Heteroscleromorpha** Cárdenas, Pérez et Boury-Esnault, 2012;

Order **Tetractinellida** Marshall, 1876;

Family **Ancorinidae** Schmidt, 1870;

Genus***Rhabdastrella*** Thiele, 1903;

***Rhabdastrella globostellata*** Carter, 1883.

The marine sponge *R. globostellata* [15] is quite widely distributed across the world’s oceans: from the East African coast to the Tonga Islands and from Taiwan to eastern Australia (Tweed–Moreton). Individuals of this species were also found in the waters of Vietnam [11,16].

**Figure 3:**
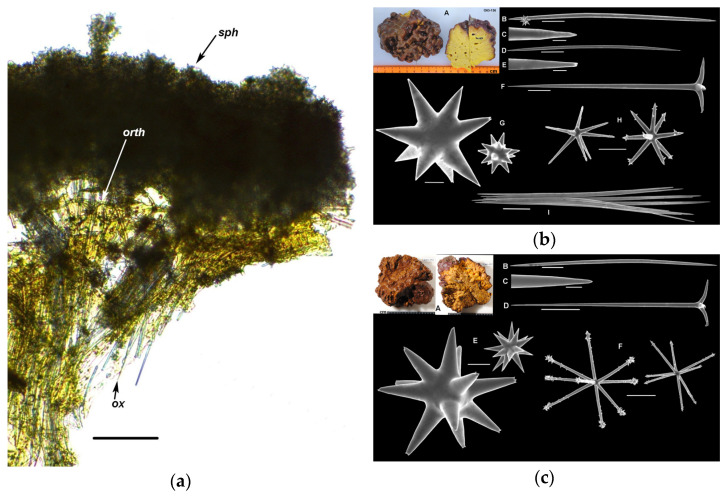
*Rhabdastrella globostellata* (**a**) Specimen PIBOC O63-136: light microscopy image of perpendicular section of skeleton. Abbreviations: sph—spheroxyasters; orth—orthotriaenes; ox—oxeas. Scale bars: 200 μm. (**b**) Specimen PIBOC O63-136: habitus of a fresh specimen (A), SEM-images of spicules, including oxeas and oxeas ends (B–E), orthotriaene (F), spheroxyasters (G), oxyasters (H), and raphides (I). Scale bars: 100 μm for B, D, and F, and 10 μm for C, E, and G–I. (**c**) Specimen PIBOC O66-092: habitus of fresh specimen (A), SEM-images of spicules, including oxea and oxea end (B, C), orthotriaene (D), spheroxyasters (E), and oxyasters (F). Scale bars: 100 μm for B and D; 10 μm for C, E, and F.

The exterior of the studied sponge specimens 1–9 was typical for *R. globostellata* (Figure 3). They were massive, with the surface being covered with multiple folds up to 1 cm high. Oscula, 1–2 mm in diameter, were located in groups in depressions on the body surface. The surface color was brown, and the interior was bright yellow, turning pale yellow in EtOH.

The sponge skeleton is presented in Figure 3. The ectosome contained a thick, 370–450 µm, layer of spheroxyasters with a small number of oxyasters. The choanosome consisted of radially arranged bundles of oxeas and orthotriaenes, the cladomes of orthotriaenes were located under the ectosome, and the rhabdomes were directed inside the sponge body. In the deeper parts of the body, bundles of oxeas and individual oxeas were arranged randomly, without a specific order. The choanosome also included spheroxyasters and oxyasters.

We examined 12 literature sources from the World Porifera Database (WPD) [16] that provide descriptions of the spicule set of *R. globostellata*. Six of them reported the presence of raphides among the spicules of *R. globostellata* [17,18,19,20,21,22], while another six do not mention raphides [11,15,23,24,25,26]. The studied sponges included both samples with raphides (PIBOC O34-077, PIBOC O49-030, PIBOC O49-055, PIBOC O63-090, PIBOC O63-136) and without them (PIBOC O49-009, PIBOC O66-089, PIBOC O66-092, PIBOC O66-109). Figure 3b,c shows images of spicules from both variants using samples PIBOC O63-136 and PIBOC O66-092; the sizes are given in Table S1. The average sizes of spicules differed insignificantly.

The morphology description for the specimen PIBOC O66-120 performed by the same methodology confirmed the *Geodia* sp. taxonomy, because the sponge exterior and skeleton were typical for the genus [27] (Figure S2).

DNA was isolated from five sponge specimens (Table 1). As a result of sequencing, we obtained three fragments of nuclear ribosomal genes for the PIBOC O63-136 specimen: a part of the 18S rRNA gene (PV055962), where PCR and sequencing were successfully achieved only with primers 560F18S/1350R18S; the end of the 18S rRNA gene and a part of the internal transcribed spacer (18S rRNA—ITS) (PV061049); and the third fragment comprising the remaining part of the ITS together with the fragment of the 28S rRNA gene (ITS—28S rRNA) (PV061050), since an “indel” was found in the sequence of the ITS.

Further comparison with the sequences available in GenBank showed that the fragment of the 18S rRNA gene (804 bp) we obtained was completely identical to KC902160 [28], KY947245, and KY947251, and differed by one nucleotide from KX894475 [29] and by two nucleotides from KY947246. All these nucleotide sequences belonged to samples of the species *R. globostellata*. Furthermore, the obtained 28S rRNA sequence was completely identical to the fragment with the accession no. AY561939 [30] over 457 nucleotides and to the sequences KC884843 [31] and ON323572 [32] over 629 nucleotides. These sequences also belonged to specimens of *R. globostellata* sponges.

For the sponge samples PIBOC O63-090, PIBOC O66-089, PIBOC O66-092, and PIBOC O66-109 we managed to obtain nucleotide sequences comprising the 18S rRNA gene without the start, ITS1, 5.8S rRNA, ITS2, and a part of the 28S rRNA gene (PV061048, PV061051, PV061052, PV061053, respectively. Table 1). The length of the sequences was 4645 bp. The ribosomal genes of the PIBOC O66-089 and PIBOC O66-092 specimens were completely identical. The sequence of the PIBOC O63-090 sample contained two nucleotides that we considered as ambiguous, and the PIBOC O66-109 specimen yielded a sequence with four such nucleotides. We compared the four obtained sequences with those presented in GenBank. We found that the 18S rRNA fragment was most similar to the 18S rRNA sequences KC902160, KY947245, KY947246, KY947251, and KX894475 provided previously for *R. globostellata* samples. The sequence similarity ranged from 99.77% to 100%. Concerning the 28S rRNA gene fragment of the studied Vietnamese specimens, the highest homology ranging from 99.69% to 99.92% was also recorded for the *R. globostellata* sequences AY561939, KC884843, and ON323572.

### 2.2. Isomalabaricanes from R. globostellata (PIBOC O63-136)

**Figure 4:**
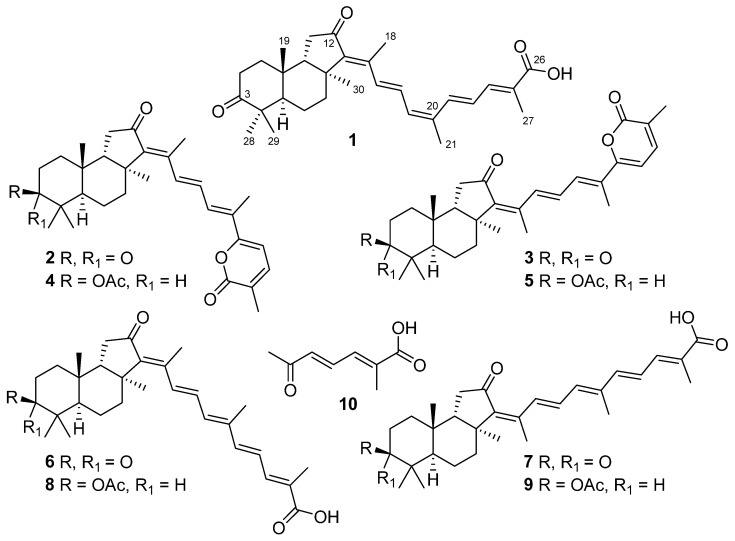
Structures of compounds **1**–**10** isolated from *R. globostellata* (PIBOC O63-136).

The concentrated EtOH extract of the marine sponge *R. globostellata* (PIBOC O63-136) was partitioned and the sum of EtOA_C_ soluble substances was separated by Sephadex LH-20 and silica gel column chromatography using different CHCl_3_/EtOH systems. For further purification by reversed-phase HPLC, we selected the major fraction E and a mid-polar fraction J because their bright yellow color was characteristic of some isomalabaricanes [1,10]. As a result, nine isomalabaricane triterpenoids **1**–**9** were isolated, including one new metabolite **1**. Also, the more polar fraction M was divided to yield compound **10** (Figure 4).

The molecular formula C_30_H_40_O_4_ of compound **1** was deduced from the peak at *m*/*z* 463.2850 [M–H]^−^ found in the (–)HRESIMS spectrum (Figure S4). Furthermore, the fragmentation ion peak at *m*/*z* 419.2943 [M–H–CO_2_]^−^ in the (–)HRESIMS/MS spectrum (Figure S4) suggested a carboxyl function in the molecule.

^13^C NMR signals of **1** (Table 2, Figure S6) showed 30 carbons, including atoms of carboxyl with *δ*_C_ 171.5, ketone with *δ*_C_ 219.0, and a ketone group at C-12 with *δ*_C_ 206.9 typical of most isomalabaricanes. Together with ^1^H NMR chemical shifts (Figure S5), they were also consistent with five double bonds in **1**: one fully substituted (*δ*_C_ 146.5 and 141.7), two trisubstituted (*δ*_H_ 6.31, d (11.6); *δ*_C_ 133.8, 136.8 and *δ*_H_ 7.45, d (11.6); *δ*_C_ 140.4, 126.9), and two disubstituted (*δ*_H_ 6.66, d (14.9) and 7.19, dd (14.9, 11.6); *δ*_C_ 133.6, 130.4 and *δ*_H_ 7.21, d (15.0) and 6.63, dd (15.0, 11.6); *δ*_C_ 135.9, 125.8). To satisfy the 11 degrees of unsaturation calculated for **1**, a tricyclic framework was required in addition to the eight double bond equivalents accounted for. The presence of seven singlets of CH_3_-18 (*δ*_H_ 2.37, s; *δ*_C_ 14.6), CH_3_-19 (*δ*_H_ 0.86, s; *δ*_C_ 23.5), CH_3_-21 (*δ*_H_ 2.06, s; *δ*_C_ 20.6), CH_3_-27 (*δ*_H_ 2.03, s; *δ*_C_ 12.7), CH_3_-28 (*δ*_H_ 1.12, s; *δ*_C_ 29.2), CH_3_-29 (*δ*_H_ 1.06, s; *δ*_C_ 19.4), and CH_3_-30 (*δ*_H_ 1.43, s; *δ*_C_ 25.9), including three olefinic ones, in the NMR spectra supported the isomalabaricane triterpenoid nature of compound **1**.

**Figure 5:**
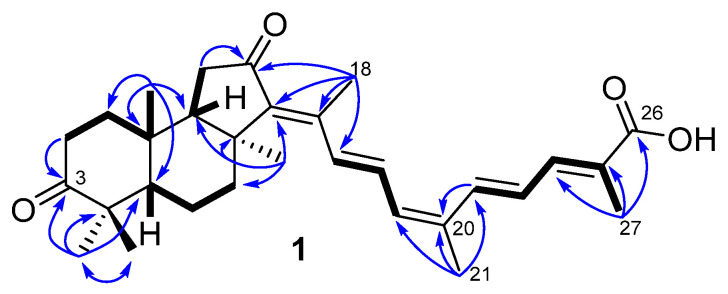
Selected COSY (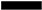) and HMBC (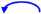) correlations of compound **1**.

The structure elucidation of **1** was completed using the 2D NMR correlation analysis (Figures S7–S10). The key HMBC correlations of methyls CH_3_-19/C-1, C-5, C-9, C-10; CH_3_-28/C-3, C-4, C-5, C-29; CH_3_-30/C-7, C-8, C-9, C-13; CH_3_-18/C-12, C-13, C-14, C-15; CH_3_-21/C-17, C-20, C-22; and CH_3_-27/C-24, C-25, C-26, and protons H_α_-2/C-3; H_2_-11/C-12; and H-22/C-20 confirmed the 3,12-diketo-6,6,5-tricyclic terpenoid core in **1** connected to a polyunsaturated side chain with terminal 26-carboxyl function (Figure 5).

**Figure 6:**
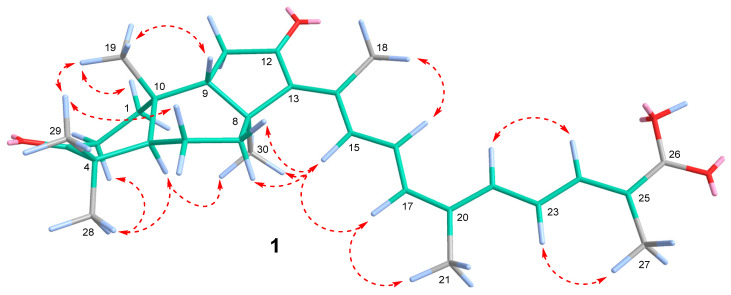
Selected ROESY (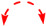) correlations of compound **1**.

According to the ROESY cross-peaks between protons H_α_-5/CH_3_-28, CH_3_-30 and CH_3_-19/H_β_-9, and CH_3_-29, compound **1** had a 5*R*,8*S*,9*S*,10*R* geometry of the tricyclic core typical for isomalabaricanes. An *E*-configured tetrasubstituted 13-double bond in **1** was deduced from the signal of CH_3_-18 (*δ*_H_ 2.37) deshielded by the 12-keto group [6], and supported with the H-15/CH_3_-30, H_α,β_-7 ROESY correlations. 15*E*,22*E*-stereochemistry led from the large vicinal coupling constants (*J*_15,16_ = 14.9 Hz and *J*_22,23_ = 15.0 Hz) measured for disubstituted double bonds. This assumption, as well as the 24*E*- and 17*Z*-configurations in **1**, were confirmed by the ROESY cross-peaks between protons H-17/H-15, CH_3_-21; H-16/CH_3_-18; and H-22/H-24, H-23/CH_3_-27 and the *W*-path COSY correlation H-24/CH_3_-27. In addition, ROESY correlations H_α_-1/H_α_-5, CH_3_-30; H_β_-1/CH_3_-19; H_α_-2/CH_3_-28; and H_β_-6/CH_3_-29 allowed assignment of the corresponding methylene protons (Figure 6).

The comparison of HRESIMS data, NMR characteristics, and optical rotations measured for **2**–**9** with the literature data led to their identification as known isomalabaricanes, rhabdastrellic acid A (**6**) [33] and stellettins A (**2**) [6,34] (^1^H and ^13^C NMR spectra in CDCl_3_ and the assigned NMR signals for **2** are presented in Figures S11 and S12, and Table S2), B (**3**) [6,34,35], C (**4**) [34,36], D (**5**) [34,36] (Figures S13 and S14, and Table S2), E (**7**) [34,37], H (**8**) [37], and I (**9**) [37]. In addition, the deduced molecular formula and the NMR signals suggested polar compound **10** to be a 2*E*,4*E*-2-methyl-6-oxo-2,4-heptadienoic acid known as a synthetic product [38] and a component of traditional Chinese herbal liquor [39]. In the *R. globostellata* extract, this compound undoubtedly represented a product of oxidative degradation of the isomalabaricane side chain. We propose that the isolation of known isomalabarican congeners from the same species *R. globostellata* allows the established relative stereochemistry of **1** to be considered the absolute configuration. Compound **1** was named as 17*Z*-rhabdastrellic acid A (17*Z*-RAA), because it was shown to be a new 17*Z*-isomer of rhabdastrellic acid A (RAA) [33].

### 2.3. LC–ESI MS Metabolomic Profiling, Chemometric Analysis, and Molecular Networking of R. globostellata Samples

The ethanolic extracts of the nine sponges identified as *R. globostellata* (Table 1) were analyzed by LC–ESI MS in the negative ion mode (Figure S15). Metabolite profiling revealed a broad diversity of secondary metabolites. A detailed analysis of the dataset with the mzMine software (ver.2.53) resulted in the detection of more than 1500 features across all the samples examined. Most of these features were distributed within the range of *m*/*z* 300–550, consistent with the masses of previously reported isomalabaricane triterpenoids and their derivatives [1,2,40]. Specifically, a comparison of retention times, high-resolution mass spectra, and MS/MS fragmentation patterns with those of previously isolated standards allowed the unambiguous identification of 16 isomalabaricanes in the analyzed samples (Table 3).

A detailed analysis demonstrated that most compounds were shared across all or several specimens. In addition, clear variations in their relative abundance were observed across individual sponges. For example, among the identified isomalabaricanes, stellettin S, globostelletin K, jaspolide F, globostelletin G, and rhabdastrellic acid A (RAA) were the predominant compounds in most samples, except for PIBOC O66-089, PIBOC O66-092, and PIBOC O66-109. In these extracts, the relative abundance of all the investigated isomalabaricane standards was reduced, except for globostelletin N, which was the dominant metabolite. To further study the patterns of similarity and to identify potential groupings among the samples, principal component analysis (PCA) and hierarchical cluster analysis were performed.

**Figure 7:**
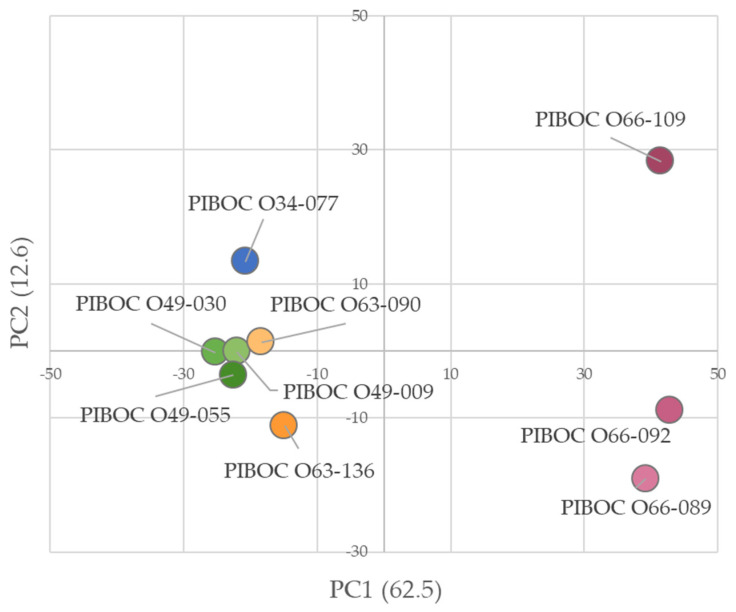
PCA score plot of metabolomic profiles for nine *Rhabdastrella* specimens, showing their projection onto the first two principal components.

The PCA revealed clear separation of the nine *Rhabdastrella* samples based on their metabolomic profiles (Figure 7). The first two principal components explained 75.1% of the total variance (PC1 = 62.5%, PC2 = 12.6%). Along PC1, three samples (PIBOC O66-089, PIBOC O66-092, and PIBOC O66-109) were distinctly segregated from all others, indicating pronounced differences in their metabolite composition. The remaining specimens formed two main patterns: a cluster of co-located specimens (PIBOC O49-009, PIBOC O49-030, PIBOC O49-055, and PIBOC O63-090) that were grouped closely together, and two individual samples, PIBOC O34-077 and PIBOC O63-136, that were separated along PC2. The clustering pattern suggested that geographic origin rather than year of collection was the primary driver of chemical variability, as exemplified by PIBOC O63-090, which was grouped with PIBOC O49-009, PIBOC O49-030, and PIBOC O49-055 from the same site but was collected in a different year (Table 1). Conversely, PIBOC O63-090 and PIBOC O63-136, although obtained during the same expedition, were positioned distantly in the PCA plot, reflecting site-specific differences.

**Figure 8:**
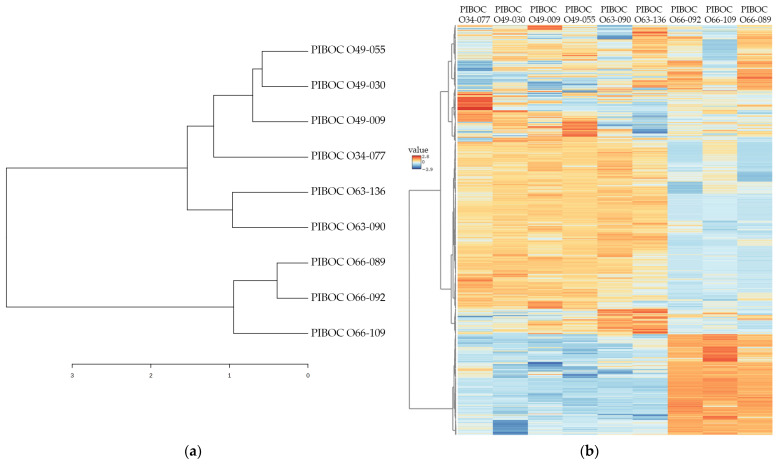
(**a**) Hierarchical clustering dendrogram of nine *Rhabdastrella* specimens based on their metabolomic profiles. (**b**) Heatmap of relative metabolite feature intensities (rows) across the nine specimens (columns), with unsupervised hierarchical clustering applied to features; colors denote relative abundance (red, higher; blue, lower).

The dendrogram (Figure 8a) and the heatmap (Figure 8b) corroborated the PCA findings by grouping samples with similar metabolomic profiles. Specimens PIBOC O66-089, PIBOC O66-092, and PIBOC O66-109 formed a tight cluster (designated below as PIBOC O66-###) that was clearly separated from the remaining specimens. In contrast, all other samples collected from sites 1–3 (Figure 1) appeared on a separate branch of the dendrogram. Within this branch, PIBOC O34-077 was closer to the PIBOC O49-### cluster, whereas PIBOC O63-090 and PIBOC O63-136 formed a distinct subcluster. The heatmap highlighted pronounced metabolomic differences between the Phan Thiet specimens (PIBOC O66-### from site 4) and all other sponges.

Since only a small number of authentic reference standards were available and MS/MS spectra of previously isolated isomalabaricanes were largely absent from public spectral libraries, only a minor fraction of the detected metabolites could be unequivocally identified. To obtain a more comprehensive view of the isomalabaricane chemical space present in the analyzed extracts, we therefore applied feature-based molecular networking (FBMN) of the LC-ESI MS/MS data on the Global Natural Products Social Molecular Networking (GNPS) platform. This approach clusters features with similar fragmentation patterns, enabling rapid visualization of metabolite diversity and structural relatedness within complex mixtures and facilitating analog discovery [43].

**Figure 9:**
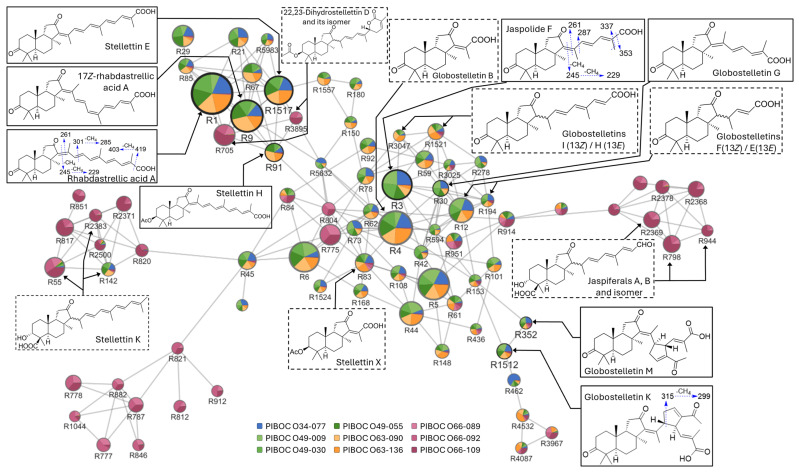
Feature-based molecular network (cluster A) from LC-ESI MS/MS data of nine *Rhabdastrella* extracts, highlighting the isomalabaricane family. Nodes represent MS/MS features; pie charts indicate the relative contribution of each sample, and edges connect nodes with similar fragmentation spectra. Boxes indicate nodes annotated to known isomalabaricanes with the corresponding chemical structures: solid line indicates unambiguously identified compounds, whereas dashed line indicates tentative annotations. Remaining labeled nodes (R###) denote putative analogs revealed by spectral similarity; unlabeled nodes represent artifactual features.

**Figure 10:**
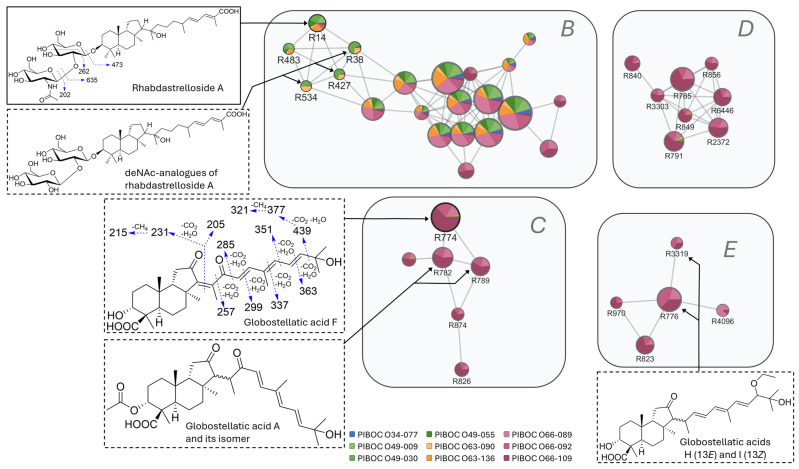
Feature-based molecular network (clusters B–E) from LC-ESI MS/MS data of nine *Rhabdastrella* extracts. Nodes represent MS/MS features; pie charts indicate the relative contribution of each sample; and edges connect nodes with similar fragmentation spectra. Boxes indicate nodes annotated to known isomalabaricanes with the corresponding chemical structures: solid line indicates unambiguously identified compound, whereas a dashed line indicates tentative annotations. Remaining labeled nodes (R###) denote putative analogs revealed by spectral similarity, unlabeled nodes represent artifactual features or non-isomalabaricane compounds.

The molecular network comprised 1505 nodes, of which 1251 were singletons (Figure S17, Table S3). Among the small local clusters, 12 contained three or four nodes, while 33 were two-node clusters. Seven larger clusters A–G contained more than five nodes; two exceeded 20 nodes and formed densely connected sub-networks enriched in isomalabaricane metabolites. A color-distribution analysis corroborated the segregation of the PIBOC O66-### samples: the nodes colored in pink hues were located almost exclusively on peripheral branches and in isolated clusters, with minimal co-localization with nodes from the remaining samples (Figure 9, Figure 10 and Figure S17).

As noted above, most of the detected features appeared as singleton nodes in the molecular network. Among the singleton nodes, several isomalabaricane metabolites were identified by comparison of retention times and MS data with those of the authentic standards. However, these metabolites did not assemble into clusters due to various factors. Specifically, the precursor ions of stellettins A, B, and S, and the globostelletin N were of unsatisfactory intensity or their MS/MS spectra lacked diagnostic product ions and were, therefore, insufficiently informative for molecular networking. Conversely, rhabdastrelloside B and stellettin W produced informative product ions, yet their fragmentation fingerprints were insufficiently similar to those of other compounds to surpass the clustering threshold. Additionally, manual inspection revealed that most singleton nodes had non-informative MS/MS spectra or low-intensity, low-quality spectra, whereas small clusters most frequently corresponded to closely related ion pairs (e.g., monomer–dimer), adducts, or in-source fragments.

The largest cluster A (Figure 9) comprised 81 nodes and represented a highly diversified yet biosynthetically coherent family of isomalabaricane metabolites. The cluster topology analysis revealed a dense core surrounded by peripheral subclusters connected to the core via a small number of bridging nodes. The core encompassed more than 50 nodes, most abundant in specimens PIBOC O34-077, PIBOC O49-009, PIBOC O49-030, PIBOC O49-055, PIBOC O63-090, and PIBOC O63-136. In the left and right sectors of cluster A, three small subclusters aggregated closely related metabolites predominantly detected in the PIBOC O66-### samples. The minimal overlap between these subclusters and the core suggested site-specific chemotypes and indicated the presence of an independent biosynthetic pathway unique to PIBOC O66-### specimens.

The central region of cluster A included dozens of metabolites exhibiting similar fragmentation patterns, thereby emphasizing their close structural relationship. Node R3 (C_25_H_34_O_4_, 13.09 min) corresponded to one of the most intense metabolites, occupying a central position in cluster A. This compound was unambiguously identified as jaspolide F because its retention time and MS data matched those of an authentic standard. In the negative ion MS/MS spectrum of jaspolide F, two abundant product ions were observed at *m*/*z* 353, arising from the neutral loss of CO_2_, and at *m*/*z* 337, formed by the sequential loss of CO_2_ and CH_4_. The fragment ion [M–H–CO_2_]^−^ confirmed the presence of a free carboxyl group. According to the literature data, the ion [M–H–CO_2_–CH_4_]^−^ is diagnostic for *α*,*β*-unsaturated acids [44]. In the case of isomalabaricanes, this fragment is most plausibly explained by the loss of CH_4_ from C-25 followed by CO_2_ elimination. Considered together, this fragmentation pattern represents a diagnostic feature of isomalabaricanes that possess an unsaturated side chain terminating in a carboxyl group. Additional product ions at *m*/*z* 287 [M–H–C_6_H_6_O_2_]^−^, 261 [M–H–C_8_H_8_O_2_]^−^, 245 [M–H–C_9_H_12_O_2_]^−^, and 229 [M–H–C_10_H_16_O_2_]^−^ originated from stepwise side-chain cleavages and corroborated both the oxidation state and the absence of further substitution in the isomalabaricane core. Three metabolites, R30, R278, and R3025, exhibited molecular formulae and fragmentation patterns similar to jaspolide F but were separated by LC where they eluted at 13.51, 13.25, and 13.16 min, respectively. Feature R30 was unambiguously identified as globostelletin G because its retention time and MS data matched those of the authentic standard. It could be hypothesized that R278 and R3025 represented isomers of jaspolide F and globostelletin G differing in the configuration of one or more double bonds within the side chain.

Among the nodes located closest to jaspolide F in the molecular network, the most intense was R4 (C_20_H_28_O_4_, 7.87 min). Its fragmentation pattern resembled that of jaspolide F, but the diagnostic ions [M–H–CO_2_]^−^ and [M–H–CO_2_–CH_4_]^−^ were shifted by −66 Da. The molecular formula together with a markedly reduced retention time (7.87 min vs. 13.09 min for jaspolide F) suggested the presence of a shortened side chain. Accordingly, compound R4 could be annotated as the known globostelletin B [4], which shared the same tricyclic core and 13*Z*-configuration with jaspolide F but differed by possessing a shortened side chain. R78 (C_20_H_28_O_4_, 8.7 min) displayed the same molecular formula and fragmentation pattern as R4 and was annotated as its unknown 13*E*-isomer. In contrast, R83 (C_22_H_32_O_5_, 11.46 min) produced diagnostic ions shifted by +44 Da relative to R4, along with additional fragment ions [M–H–CO_2_–C_2_H_2_O]^−^ and [M–H–CO_2_–C_2_H_4_O_2_]^−^. These data were consistent with the structure of recently isolated stellettin X [14] bearing a 3-OAc function. The longer retention time supported this assignment, as 3-*O*-acetylation is known to increase chromatographic retention. For instance, the 3-OAc function in stellettin H extends its retention by 3.2 min relative to the non-acetylated analog RAA (Table 3).

The isomeric metabolites R12 and R194 (C_22_H_30_O_4_, 10.4 and 10.78 min, respectively) showed strong structural similarity to jaspolide F but exhibited a 40 Da lower mass. This mass difference, considered together with their closely related product-ion spectra, suggested the presence of a side chain containing five carbon atoms. Two 13*Z*/*E* isomers consistent with this structural characteristic, globostelletins F and E [4], were previously isolated. In accordance with the observation that jaspolide F (R3) with a 13*Z*-geometry displayed the shortest retention time among 13*E* and other isomers (R30, R278, and R3025), a comparable elution order was expected for the shortened congeners. Therefore, tentative assignment of R12 to globostelletin F (13*Z*) and R194 to globostelletin E (13*E*) was proposed. Metabolite R153 (C_22_H_32_O_4_, 9.6 min), which is positioned topologically close to R12 in the molecular network, exhibited a molecular formula differing by two additional hydrogen atoms. Its fragmentation pattern was similar to that of R12, but all product-ion *m*/*z* values were shifted by +2. These data were most consistent with a core modification, presumably the replacement of the 3-keto group in R12 with a hydroxyl substituent in R153.

Two isomeric metabolites R42 (12.33 min) and R594 (12.45 min), which produced identical MS/MS spectra, shared the molecular formula C_25_H_36_O_4_, containing two additional hydrogen atoms relative to that of jaspolide F. This mass difference entirely corresponded to the reduction of a double bond. Indeed, the diagnostic product ions arising from the neutral losses of CO_2_ and CO_2_+CH_4_ appeared by 2 Da higher than the corresponding peaks in the spectrum of jaspolide F. However, the fragment at *m*/*z* 287, generated by cleavage of the C_14,15_-bond, was invariant across all three compounds. Therefore, we proposed R42 and R594 to be 15,16-dihydro analogs of jaspolide F. In a similar manner, feature R61 (C_20_H_30_O_4_, 6.79 min) was tentatively annotated as a dihydro-congener or 3-hydroxy-analog of globostelletin B (R4). This assertion was corroborated by a 2 Da mass increment and the concomitant +2 Da shifts in the product-ion series.

In the MS/MS of the isomeric features R1521 and R3047 (C_27_H_36_O_4_, 14.12 and 14.25 min, respectively), abundant product ions were observed at *m*/*z* 379 [M–H–CO_2_]^−^ and 363 [M–H–CO_2_–CH_4_]^−^, which was consistent with the presence of a terminal carboxyl group. An additional fragment ion was detected at *m*/*z* 349 [M–H–CO_2_–C_2_H_6_]^−^, accompanied by characteristic series at *m*/*z* 287, 261, and 245, that matched the side-chain fragmentation pattern of jaspolide F. The similarity of these side-chain fragments indicated that the higher precursor mass was best explained by a two-carbon elongation of the side chain. Considering the elution order, R1521 might be annotated as globostelletin I, while R3047 was presumably globostelletin H.

In the lower central sector of cluster A, a number of previously unreported nitrogen-containing metabolites was detected (features R44, R101, R148, and R436). Each feature exhibited an even nominal mass of precursor ions with elemental compositions C_22_H_31_NO_4_ (R44), C_22_H_33_NO_5_ (R101), C_22_H_31_NO_5_ (R148), and C_22_H_35_NO_5_ (R436). Their MS/MS spectra were mutually similar, showing the diagnostic product ions [M–H–CO_2_]^−^ and [M–H–CO_2_–CH_4_]^−^ characteristic of a terminal carboxyl group in an unsaturated side chain, along with a distinctive pair of fragments arising from the combined neutral loss of CO_2_ and a 57.02 Da moiety (C_2_H_3_NO): [M–H–CO_2_–C_2_H_3_NO]^−^ and [M–H–CO_2_–CH_4_–C_2_H_3_NO]^−^. To date, there is no data on nitrogen-containing metabolites from *R. globostellata* except for the recently found isomalabaricane hainanstelletin A [45]. Accordingly, these features could not be confidently annotated in the present study but they represented intriguing candidates for future bioprospecting.

Features R352 and R1512 (C_30_H_40_O_5_, 12.11 and 12.28 min, respectively) were unambiguously identified as isomeric globostelletins M and K, respectively, as their retention times and MS data matched those of the authentic standards. The MS/MS spectra of both metabolites were characterized by the presence of an intense, highly specific pair of product ions at *m*/*z* 315 [C_21_H_31_O_2_]^−^ and 299 [C_20_H_27_O_2_]^−^ produced by the cleavage of the C_15,23_ and C_16,17_ bonds of the cyclopentene unit. Isomeric features R3967 and R4532 (C_30_H_42_O_6_, 11.66 and 11.99 min, respectively) displayed a fragmentation pattern closely related to that of globostelletins K/M, but the dominant side-chain cleavage ions were shifted to *m*/*z* 301 [C_20_H_29_O_2_]^−^ and 285 [C_19_H_25_O_2_]^−^. Together with the increase in the precursor masses by +18 Da relative to globostelletins K/M, these shifts were best explained by additional oxygenation of the side chain (e.g., 17-hydroxylation as in the structure of previously isolated globostelletin J [41]). Feature R4087 (C_30_H_40_O_6_, 12.36 min) yielded an MS/MS spectrum highly similar to those of R3967/R4532, but its precursor ion was 2 Da lower in mass, indicating an additional unsaturation in the side chain while retaining the same cleavage manifold. Feature R462 (C_29_H_38_O_5_, 13.64 min) exhibited the same intense, highly specific product ions at *m*/*z* 301 and 285, whereas its molecular formula indicated a shortened side chain relative to R3967/R4532.

Cluster A also contained several other features that exhibited diagnostic fragmentation patterns, supporting their tentative structural assignments. Features R62, R59, R73, R168, R1524, and R5632 displayed the product ions [M–H–CO_2_]^−^ and [M–H–CO_2_–CH_4_]^−^, consistent with the presence of an unsaturated side chain terminating in a free carboxyl group. In contrast, the MS/MS spectra of features R5, R108, R775, R804, R914, and R951 retained the [M–H–CO_2_]^−^ product ion but also exhibited the neutral losses H_2_O and CO_2_ + H_2_O, while lacking the [M–H–CO_2_–CH_4_]^−^ fragment. Finally, features R6, R45, R84, and R92 generated a distinctive CO-loss series, comprising [M–H–CO]^−^, [M–H–CO_2_]^−^, and [M–H–CO−CO_2_]^−^. The observation of CO-loss is indicative of the presence of a carbonyl group [44]. The limited experimental data, combined with the absence of relevant reports in the literature, prevents confident structural assignment of these compounds.

The three most abundant features in the upper part of cluster A—nodes R1 (C_30_H_40_O_4_, 16.87 min), R9 (C_30_H_40_O_4_, 17.27 min), and R1517 (C_30_H_40_O_4_, 17.37 min)—exhibited identical product-ion spectra and were unambiguously identified by comparison with authentic standards such as rhabdastrellic acid A (RAA), 17*Z*-rhabdastrellic acid A (**1**) (17*Z*-RAA), and stellettin E (**7**), respectively. These isomers shared an isomalabaricane core and a highly unsaturated side chain, differing only in the configurations of the double bonds. MS/MS spectra of these compounds were very similar and displayed a fragmentation pattern characteristic of the isomalabaricane class. For example, in the MS/MS spectrum of RAA (R1), prominent product ions were observed at *m*/*z* 419 [M–H–CO_2_]^−^ and 403 [M–H–CO_2_–CH_4_]^−^, confirming the presence of an unsaturated side chain terminating in a free carboxyl group. The side-chain cleavage of C_15,16_-bond yielded product ions at *m*/*z* 301 [M–H–C_10_H_10_O_2_]^−^ and 285 [M–H–C_10_H_10_O_2_–CH_4_]^−^, whereas fragmentation across the C_13,14_-bond produced fragments at *m*/*z* 261 [M–H–C_13_H_14_O_2_]^−^, 245 [M–H–C_13_H_14_O_2_–CH_4_]^−^, and 229 [M–H–C_13_H_14_O_2_−2CH_4_]^−^. Additional metabolites R21 (16.24 min), R85 (16.58 min), and R5983 (16.38 min) showed molecular masses and fragmentation patterns analogous to those of RAA and eluted at comparable retention times. These findings suggested that the above listed minor congeners possess closely related structures and differed from RAA only in the *E*/*Z* configuration of one or more side-chain double bonds.

Two nodes, R29 and R67, which were topologically adjacent to the RAA group, exhibited elemental compositions containing two additional hydrogen atoms relative to RAA. Their fragmentation patterns resembled that of RAA but showed informative differences. For R67 (C_30_H_42_O_4_, 16.29 min), all side-chain cleavage ions were shifted by +2 Da, indicating that the mass increment was localized in the triterpenoid core and was most consistent with the replacement of the 3-keto group by a hydroxyl substituent. Accordingly, R67 might be annotated as isomeric stellettins M or L [46]. In contrast, the MS/MS spectrum of R29 (C_30_H_42_O_4_, 16.8 min) displayed shifts by +2 Da only in the product ions [M–H–CO_2_]^−^ and [M–H–CO_2_–CH_4_]^−^, whereas the side chain cleavage series at *m*/*z* 301, 285, 261, 245, and 229 was identical to that of RAA, indicating that hydrogenation was confined to the 22- or 24-double bond of the side chain.

The feature R91 (C_32_H_44_O_5_, 20.03 min) was identified as stellettin H based on comparison of retention time and MS data with an authentic standard. The fragmentation pathways of stellettin H resembled those of RAA. However, two parallel fragmentation series, separated by 44 Da, were observed. Together with a 3.2 min increase in retention time, this observation indicated the presence of an acetoxy substituent at C-3. Features R3895 (19.75 min) and R705 (19.89 min) shared the same elemental composition as R91 and eluted at similar retention times, but their negative ion MS/MS spectra were largely uninformative. This behavior suggested that, in contrast to R91, both metabolites lacked a free carboxyl and/or hydroxyl group capable of stabilizing the deprotonated precursor and enabling informative fragmentation, which explained the paucity of product ions. Esterification or intramolecular lactonization could plausibly account for the absence of a charge-bearing site. Due to their proximity to stellettin H, metabolites R3895 and R705 might be considered as structural analogs of the known 22,23-dihydrostellettin D [47]. They probably differed only in the *E*/*Z*-configuration of one or more side chain double bonds. Notably, stellettin H (R91) was observed in PIBOC O34-077, PIBOC O49-009, PIBOC O49-030, PIBOC O49-055, PIBOC O63-090, and PIBOC O63-136, whereas R3895 and R705 were characteristic of the PIBOC O66-### series.

Metabolites R150 (15.08 min), R180 (14.51 min), and R1557 (14.28 min) exhibited the same elemental composition (C_31_H_39_NO_5_) and identical MS/MS spectra. The product ions [M–H–CO_2_]^−^ and [M–H–CO_2_–CH_4_]^−^ corresponded to an unsaturated side chain with a terminal carboxyl group, while the additional ion [M–H–CO_2_−H_2_O]^−^ suggested the presence of at least one hydroxyl substituent. The even nominal mass of the [M–H]^−^ precursors, together with the intense fragment ions [C_13_H_12_NO]^−^ and [C_11_H_10_NO]^−^ supported the presence of a single nitrogen atom in the molecule. On the other hand, the spectra displayed a characteristic side-chain cleavage series at *m*/*z* 301, 285, 261, and 245, indicative of conservation of the RAA-type isomalabaricane core.

The right-hand subcluster comprised metabolites observed exclusively in the PIBOC O66-### specimens (Figure 9). The analysis revealed three isomeric metabolites R798, R944, and R2369 with the elemental formula C_27_H_36_O_5_ and two compounds R2368 and R2378 characterized by the formula C_29_H_38_O_6_. Their fragmentation pathways differed markedly from those grouped in the central sector of cluster A. The isomeric features R798 (10.98 min), R944 (10.75 min), and R2369 (10.86 min) produced product ions at *m*/*z* 421 [M–H−H_2_O]^−^, 411 [M–H–CO]^−^, 395 [M–H–CO_2_]^−^, 377 [M–H–CO_2_−H_2_O]^−^, 359 [M–H–CO_2_−2H_2_O]^−^, and 343 [M–H–CO_2_−2H_2_O−CH_4_]^−^, along with ions at *m*/*z* 269 [C_19_H_25_O]^−^, 257 [C_18_H_25_O]^−^, 215 [C_15_H_19_O]^−^, and 183 [C_13_H_11_O]^−^. In contrast to the central cluster metabolites, in which the peaks of fragments [M–H–CO_2_]^−^ and [M–H–CO_2_–CH_4_]^−^ typically prevailed in the MS/MS spectra, the present spectra were dominated by the [M–H–CO_2_−H_2_O]^−^ product ion, while the fragment [M–H–CO_2_–CH_4_]^−^ was absent. The low-intensity fragment [M–H–CO]^−^ was consistent with the presence of a terminal carbonyl function in the side chain. Considered together with the literature data, the lack of fragments diagnostic for a side-chain carboxyl group and the presence of the series [M–H−H_2_O]^−^, [M–H–CO_2_]^−^, and [M–H–CO_2_−H_2_O]^−^ suggested that these metabolites possessed a hydroxyl substituent at C-3 and a carboxyl group at C-4. Accordingly, features R798, R944, and R2369 were proposed to share the structural framework of jaspiferals A and B [48], differing only in the configuration of side-chain double bonds. Features R2368 (14.09 min) and R2378 (13.81 min) exhibited a closely similar fragmentation pattern. However, their elemental composition, the absence of the [M–H−H_2_O]^−^ fragment, and the retention times increased by ~3 min indicated a closely related structure with an acetoxy substituent at C-3.

Two subclusters on the left side of cluster A also grouped metabolites characteristics, mainly of the PIBOC O66-### specimens (Figure 9). The upper subcluster was distinguished by a specific fragmentation signature. The MS/MS spectrum of its main node, R55 (C_30_H_42_O_4_, 17.11 min), displayed product ions at *m*/*z* 447 [M–H−H_2_O]^−^, 421 [M–H–CO_2_]^−^, 419 [M–H–CH_2_O_2_]^−^, 403 [M–H–CO_2_−H_2_O]^−^, and 387 [M–H–CO_2_−H_2_O−CH_4_]^−^, along with ions at *m*/*z* 359 [C_22_H_31_O_4_]^−^, 297 [C_21_H_29_O]^−^, 269 [C_19_H_25_O]^−^, 257 [C_18_H_25_O]^−^, 215 [C_15_H_19_O]^−^, and 183 [C_13_H_11_O]^−^. Notably, although the above-described features, R29 and R67, shared the same elemental formula with R55, their MS/MS spectra differed: in the case of R29 and R67, the two dominant peaks corresponded to losses of CO_2_ and CO_2_+CH_4_, whereas for R55 the ion [M–H–CO_2_–CH_4_]^−^ was absent, [M–H–CO_2_]^−^ was of low intensity, and [M–H–CO_2_−H_2_O]^−^ constituted the base fragment peak. Moreover, the low-mass series at *m*/*z* 269, 257, 215, and 183 reproduced the series observed in the MS/MS spectra of R798 from the right-hand subcluster. Collectively, these data supported the presence in R55 of a 3-hydroxyl substituent and a carboxyl group at C-4, in addition to an unsubstituted polyunsaturated side chain. This assumption was further corroborated by the low-intensity fragment ion at *m*/*z* 359 [C_22_H_31_O_4_] ^−^, formed by C_17,20_-cleavage. According to the available literature data, R55, as well as its isomeric forms R142 (17.25 min) and R2383 (17.0 min), can be tentatively identified as structural analogs of stellettin K [49] or (13*Z*,15*E*,17*E*,22*E*)-3*β*-hydroxy-12-oxomalabarica-13,15,17,22,24-pentaen-28-oic acid [35,50]. All remaining members of this subgroup (R817, R820, R851, R2371, R2500) exhibited the same diagnostic fragmentation pattern, indicating a common 3-hydroxy-28-carboxy-isomalabaricane core, whereas the observed variation in precursor masses and retention times suggested differences confined to the side-chain architecture.

The small lower subcluster (R777, R778, R787, R812, R821, R846, R882, R912, and R1044) was distinguished by a short retention times and a high degree of oxidation despite relatively low molecular masses. These metabolites displayed a distinctive fragmentation fingerprint. For example, in the MS/MS spectrum of R787 (C_21_H_30_O_8_, 7.02 min), product ions were observed at *m*/*z* 349 [M–H–C_2_H_4_O_2_]^−^, 305 [M–H–C_2_H_4_O_2_–CO_2_]^−^, 287 [M–H–C_2_H_4_O_2_–CO_2_−H_2_O]^−^, and 261 [M–H–C_2_H_4_O_2_−2CO_2_]^−^, along with fragments at *m*/*z* 243 [C_17_H_23_O]^−^ and 217 [C_15_H_21_O]^−^. The high intensity of the peak [M–H–C_2_H_4_O_2_–CO_2_]^−^ was consistent with the 3-OAc group and COOH at C-4 position, whereas the additional CO_2_ elimination, accompanied by the [M–H–C_2_H_4_O_2_−2CO_2_]^−^ fragment, suggested the presence of a second carboxyl function.

Considering the pool of identified and putatively annotated metabolites that constituted cluster A, the data suggested a divergence in biosynthetic processes between the two groups of specimens. Extracts of PIBOC O34-077, PIBOC O63-090, PIBOC O63-136, PIBOC O49-009, PIBOC O49-030, and PIBOC O49-055 were enriched in structures that shared a recurring motif: a lightly oxidized isomalabaricane core in which C-3 most frequently bore a carbonyl function, and a side chain of variable lengths and unsaturation degrees terminating in a free carboxyl group. In contrast, the PIBOC O66-### set was characterized by the prevalence of structures featuring a highly oxidized core with a carboxyl group at C-4 and either a 3-hydroxy or a 3-acetoxy substituent, whereas the side chain, although being variable in length, was comparatively less oxidized or non-oxidized (Figure 9).

Cluster B comprised metabolites that were detected in all the analyzed samples (Figure 10). A distinguishing feature of these metabolites was the presence in their MS/MS spectra of product ions arising from neutral losses of C_6_H_10_O_5_. Feature R14 (C_44_H_73_NO_14_, 9.53 min) was identified as rhabdastrelloside A by comparison of its retention time and MS data with those of an authentic standard. Its MS/MS spectrum exhibited an ion series that was indicative of a stepwise loss of monosaccharide units. The presence of Y-type ions at *m*/*z* 635 [M–H–C_8_H_13_NO_5_]^−^ and 473 [M–H–C_8_H_13_NO_8_−C_6_H_10_O_5_]^−^ was consistent with the sequential neutral loss of a hexoseNAc (HexNAc) and a hexose (Hex) unit. Additional fragments were detected at *m*/*z* 202 [C_8_H_12_NO_5_]^−^, which corresponded to a deprotonated HexNAc residue, and 262 [C_10_H_16_NO_7_]^−^, which was explained by A-type cross-ring cleavage of hexose. Metabolite R483 (C_44_H_73_NO_14_, 9.33 min) showed the same molecular formula and an analogous fragmentation pattern, indicating that it represented an isomer of rhabdastrelloside A. The isomeric features R38, R427, and R534 (C_42_H_70_O_14_, 9.69, 9.89, and 9.51 min, respectively) displayed closely related retention times and fragmentation patterns. Their MS/MS spectra revealed intense Y-type product ions at *m*/*z* 635 [M–H–C_6_H_10_O_5_]^−^ and 473 [M–H−2×C_6_H_10_O_5_]^−^, which confirmed the stepwise loss of two monosaccharide residues. In contrast to R14 and R483, no HexNAc neutral loss was observed, indicating the absence of this residue in the structures of R38/R427/R534. Consequently, these metabolites were annotated as analogs of rhabdastrelloside A with a new type of disaccharide moiety.

The remaining constituents of cluster B comprised a dense module of closely related species in the molecular network, with nearly all nodes detected across all samples (Figure 10). Their MS/MS spectra exhibited a distinctive fingerprint characterized by the product ions [M–H–C_6_H_10_O_5_]^−^ and [M–H–C_6_H_12_O_6_]^−^ together with phosphate-headgroup fragments at *m*/*z* 259 [C_6_H_12_O_9_P]^−^, 241 [C_6_H_10_O_8_P]^−^, and 223 [C_6_H_8_O_7_P]^−^. This fragmentation pattern was diagnostic of phosphatidylinositol-type lipids [51,52]. The precursor-ion mass range and consistent headgroup chemistry supported tentative assignment of these metabolites as unusual ether-linked analogs of lysophosphatidyl-inositols [53] that differed in the length and degree of unsaturation of the alkyl chains.

Clusters C, D, and E included only compounds that were found exclusively in the set of PIBOC O66-### specimens (Figure 10). The most intense node R774 (8.41 min) in cluster C corresponded to a metabolite with a molecular formula of C_30_H_42_O_6_. Its MS/MS spectrum displayed diagnostic fragment ions at *m*/*z* 479 [M–H−H_2_O]^−^, 453 [M–H–CO_2_]^−^, 451 [M–H–CH_2_O_2_]^−^, and 435 [M–H–CO_2_−H_2_O]^−^, consistent with the presence of a 3-hydroxy substituent and a carboxyl group at C-4. Additional product ions at *m*/*z* 439 [M–H–C_3_H_6_O]^−^, 417 [M–H–CO_2_−2H_2_O]^−^, 395 [M–H–C_3_H_6_O−CO_2_]^−^, and 377 [M–H–C_3_H_6_O−CO_2_−H_2_O]^−^ supported the presence of an additional hydroxyl substituent and were consistent with a terminal hydroxyl group in the side chain. A homologous series of side-chain cleavages accompanied by neutral losses of CO_2_+H_2_O yielding ions at *m*/*z* 363 [C_25_H_31_O_2_]^−^, 351 [C_24_H_31_O_2_]^−^, 337 [C_23_H_29_O_2_]^−^, 321 [C_22_H_25_O_2_]^−^, 299 [C_20_H_27_O_2_]^−^, 285 [C_19_H_25_O_2_]^−^, 257 [C_18_H_25_O]^−^, 231 [C_16_H_23_O]^−^, and 215 [C_15_H_19_O]^−^, together with a fragment ion at *m*/*z* 205 [C_13_H_17_O_2_]^−^, suggested the presence of a carbonyl substituent at C-15. Collectively, these observations indicated that feature R774 most likely possessed a structure reported previously as globostellatic acid F [54]. The isomeric features of R782 and R789 (C_32_H_44_O_7_, 11.18 and 11.53 min, respectively) exhibited an MS/MS spectra closely similar to those of R774. However, the subtle differences in their elemental composition and fragmentations, combined with a ~3 min increase in retention time, were consistent with related structures bearing an acetoxy substituent at C-3. We suggest that these two features represent isomers of the known globostellatic acid A [55]. Compounds R826 (C_30_H_42_O_7_, 7.49 min) and its acetylated derivative R874 (C_32_H_44_O_8_, 10.24 min) displayed a similar behavior, indicating structures similar to those of R774 and R782/R789, respectively, but with an additional oxygenated substituent in the core. This interpretation was supported by their molecular formulae, as well as by a +16 Da shift in the main fragment-ion series, including core fragment ions at *m*/*z* 247 [C_16_H_23_O_2_]^−^ and 231 [C_15_H_19_O_2_]^−^ (rather than 231 [C_16_H_23_O]^−^ and 215 [C_15_H_19_O]^−^ in R774, R782, and R789).

Several isomeric metabolites (R785, R849, R856, R2372, R3303, and R6446) with the molecular formula C_30_H_44_O_6_ were detected within cluster D. They showed fragmentation behavior resembling that of the metabolites from cluster C. The MS/MS spectra of the dominant metabolite R785, as well as the other isomers, displayed similar product ions at *m*/*z* 481 [M–H−H_2_O]^−^, 463 [M–H−2H_2_O]^−^, 441 [M–H–C_3_H_6_O]^−^, 437 [M–H–CO_2_−H_2_O]^−^, 423 [M–H–C_3_H_6_O−H_2_O]^−^, 397 [M–H–C_3_H_6_O−CO_2_]^−^, and 379 [M–H–C_3_H_6_O−CO_2_−H_2_O]^−^. This pattern suggested the presence of a 3-hydroxy substituent, a carboxyl group at C-4, and a terminal hydroxyl group in the side chain. However, the series derived from side-chain cleavages differed from that of cluster C metabolites: fragments were observed at *m*/*z* 411 [C_26_H_35_O_4_]^−^, 397 [C_26_H_37_O_3_]^−^, 357 [C_22_H_29_O_4_]^−^, and 333 [C_20_H_29_O_4_]^−^, alongside intense peaks at 271 [C_19_H_27_O]^−^, 255 [C_18_H_23_O]^−^, 231 [C_16_H_23_O]^−^, and 215 [C_15_H_19_O]^−^. This divergence allowed us to propose a hydroxy group in an undetermined position of the side chain for R785 instead of the 15-keto function in R774. Consistent with chromatographic behavior and MS/MS fragmentation pattern, compound R840 (C_32_H_46_O_7_, 10.32 min) was annotated as the 3-*O*-acetylated derivative of R785. The MS/MS fragmentation of dehydrogenated analog R791 (C_30_H_42_O_6_, 8.86 min) closely resembled that of R785; however, a neutral loss of C_3_H_4_O was observed for several product ions instead of C_3_H_6_O in R785.

The MS/MS spectra of isomeric compounds R823 and R970 (C_30_H_44_O_6_, 8.15 and 7.93 min, respectively), which were found in cluster E, displayed product ions at *m*/*z* 481 [M–H−H_2_O]^−^, 437 [M–H–CO_2_−H_2_O]^−^, 419 [M–H–CO_2_−2H_2_O]^−^, and 403 [M–H–CO_2_−2H_2_O−CH_4_]^−^, together with core fragment ions at *m*/*z* 231 [C_16_H_23_O]^−^ and 215 [C_15_H_19_O]^−^. These data were in agreement with a core part having a hydroxyl group at C-3 and a carboxyl group at C-4. In contrast to metabolites in clusters C and D, the ion [M–H–C_3_H_6_O]^−^, diagnostic of a terminal hydroxyl group, was absent, which indicated a different oxidation pattern in the side chain. Considered together, these findings suggested that R823 had two oxygenated substituents within the side chain, although their exact positions could not be determined from the MS data alone. Feature R4096 (C_31_H_46_O_6_, 10.51 min) contained an additional oxygen atom in the core compared to R823, as indicated by the consistent +16 Da shift in the fragment-ion series and the appearance of core fragment ions at *m*/*z* 247 [C_16_H_23_O_2_]^−^ and 231 [C_15_H_19_O_2_]^−^. Features R776 and R3319 (C_32_H_48_O_6_, 11.57 and 11.36 min, respectively) demonstrated the same core scaffold as that in R823, while their molecular formulas, retention time shifts, and the presence of additional fragments [M–H–C_2_H_4_O]^−^ and [M–H–CO_2_−H_2_O−C_2_H_6_O]^−^ indicated the presence of an ethoxy group in their structures. Based on the obtained data, R3319 and R776 were tentatively assigned to structures analogous to globostellatic acids H and I [54].

The remaining clusters in the feature-based molecular network comprised either artifactual components (dimers, adducts, or in-source fragments), non-isomalabaricane compounds or unannotated features.

## 3. Discussion

Marine sponges are known as the most prolific marine sources for discovery of novel bioactive compounds. Sponge secondary metabolites are sought-after for their potential in pharmaceutical applications [56]. Terpenoids, among other metabolites, are fundamental in the biochemical defense of sponges and other sessile marine organisms. They are used in the competition for space, for deterring predators, and for protection from parasites and pathogenic microorganisms. The wide range of ecological functions of these metabolites is also reflected in their multifaceted bioactivity [57].

In the past, some natural products from sponges were used as taxonomic markers alongside the intriguing and homoplasy-prone morphology challenging species delineation. Understanding the phylogenetic distribution and specificity of metabolites to sponge lineages is essential for resolving the production pathways and evolution of these compounds in sponges. This benefits the discovery rate and yield of bioprospecting novel marine natural products through identification of sponge lineages as new sources of valuable compounds with high biological activities [56].

In particular, isomalabaricane triterpenoids were, for a long time, considered as metabolites specific to four genera of marine sponges belonging to the order Tetractinellida [1,2,3]. However, a recent revision of the reported isomalabaricane sources concluded that triterpenoids of this structural class had been found almost exclusively in samples of *R. globostellata*, one unidentified *Rhabdastrella* sample, and one putative *Geodia* sp. specimen [11]. This substantial conclusion was based on archival taxonomic descriptions and images of sponges collected from tropical and subtropical waters (from the South Pacific to Mage Shima Island, Japan, and the South China Sea), as well as data of a recent morphological analysis of the sponge samples that the authors accessed. Our present study focused on the search of isomalabaricane-containing sponges from the Vietnam waters of the South China Sea and their reliable taxonomic identification based on an integrative approach combining morphological examination and molecular analysis. As a result, all nine found isomalabaricane sources were undoubtedly identified as *R. globostellata* sponges. In addition, the specimens belonging to the genus *Geodia* did not show the presence of target triterpenoids during the chemical screening (Figure 2).

To confirm the presence of isomalabaricanes in the screened sponges, we isolated ten individual components of the EtOH extract of *R. globostellata* (PIBOC O63-136) including a new minor 17*Z*-isomer of rhabdastrellic acid A **1**, eight known isomalabaricanes **2**–**9**, and compound **10** suggested to be a product of oxidative degradation of the isomalabaricane side chain. A structural elucidation of 17*Z*-rhabdastrellic acid A (**1**) using HRESIMS and NMR experiments showed it to be an isomalabaricane triterpenoid with a new type of polyene side chain. The same 13*E*,15*E*,17*Z*,22*E*-configuration, except for the additional 24*E*-geometry in **1**, was established previously only for the pair of 13*Z*/*E*-isomeric globostellatic acid X methyl esters [58].

The chemical diversity of sponges may be useful for addressing fundamental issues in systematics or evolutionary biology, while metabolic fingerprints as indicators of metabolomic diversity can resolve interspecific and intraspecific relationships [59,60]. However, there is a limited number of reports where attempts were made to assess the production of isomalabaricanes by HPLC profiling [61] or LC–MS methods [62]. Unfortunately, those studies suggested to comprise some soft spots like a possible misidentification of the isomalabaricane-containing sponge *Stelletta tenuis* Lindgren [61] or a doubtful and completely unconfirmed hypothetical origin of red algal peyssobaricanosides A−C via rearrangement of the isomalabaricane carbon skeleton [62].

Annotation of the data obtained by LC–ESI MS profiling of the EtOH extracts of the nine *R*. *globostellata* specimens using a spectral library of the series of individual standards provided a comprehensive overview of the isomalabaricane metabolome in sponges collected from four different localities for a period from 2007 to 2023. The LC-MS data revealed a remarkable chemical diversity with more than 1500 features detected across all samples, but only 16 compounds were definitely identified by comparing their retention times, high-resolution mass spectra, and MS/MS fragmentation patterns with those of previously isolated metabolites (Table 3). This finding highlighted both the metabolic richness of the species and the limitations of available reference data for marine triterpenoids. High-resolution MS and MS/MS spectra still enabled the tentative annotation of numerous congeners that differed by subtle modifications in the core scaffold or side chain. Thus, LC-MS profiling served as a foundation for subsequent chemometric and molecular networking analyses to assess the extent of chemical diversity and intraspecific variability of the detected metabolites.

Despite a conservative range of isomalabaricane triterpenoids detected across all nine sponges, clear differences in the relative abundance of several identified metabolites were observed, ultimately revealing distinct chemotypes. The Phan Thiet specimens (PIBOC O66-### from site 4) were noticeably characterized by a depletion of rhabdastrellic acid A, jaspolide F, and stellettin series, thus indicating a unique metabolomic signature. A multivariate analysis, including PCA and hierarchical clustering, demonstrated that the specimens collected from the same locality exhibited more similar metabolomic profiles and tended to cluster together in the chemical space compared to those collected from different localities. Overall, the multivariate analysis segregated the examined *Rhabdastrella* specimens into discrete groups with distinct metabolomic signatures, revealing two primary chemotypes: the PIBOC O66 cluster, dominated by a unique suite of high-abundance metabolites not detected elsewhere, and a cluster of samples collected from sites 1–3, which shared a broadly conservative repertoire of characteristic compounds.

To obtain a more comprehensive view of the isomalabaricane chemical space present in the analyzed extracts, we applied a feature-based molecular networking (FBMN) workflow. This approach not only corroborated the results of multivariate analysis but also provided structural context to the observed diversity, enabling visualization of the relationships between known and putatively annotated isomalabaricanes. The topology of the largest network component, cluster A, revealed a multitude of structurally related metabolites forming a dense core of closely related isomalabaricanes dominating in the samples from sites 1–3, whereas metabolites characteristic of site 4 were grouped into peripheral subclusters with minimal overlap.

In addition, the network topology facilitated the recognition of structural series and allowed for the putative annotation of numerous analogs differing in side-chain oxidation, double-bond geometry, or core substitutions. As regards the pool of identified and tentatively annotated metabolites that comprised cluster A, sponges from sites 1–3 shared a recurring structural motif: a lightly oxidized isomalabaricane core together with an unsaturated side chain of variable length terminating in a free carboxyl group. In contrast, the PIBOC O66-### specimens were enriched in structures with a more highly oxidized core, characterized by a carboxyl group at C-4 and either a 3-hydroxy or 3-*O*-acetyl substituent, while their side chains were comparatively less oxidized or even unsubstituted. Moreover, the results indicate the presence of previously unreported metabolites, including not only isomers of known isomalabaricanes but also novel entities. The most promising finding was the detection of nitrogen-containing isomalabaricanes in PIBOC O66-### sponges, represented so far by one known compound [45], as well as new members of extremely rare isomalabaricane glycosides [13].

Taken together, the applied approaches not only enabled rapid dereplication of the known metabolites but also provided a framework for the discovery of novel congeners and for resolving intraspecific chemical variations in complex sponge metabolomes.

To conclude, it should be noted that isomalabaricanes have attracted the attention of natural product chemists for decades [1], but the origin of these triterpenoids in marine sponges still remains unknown. Our experimental results have confirmed that, among sponges inhabiting Vietnamese waters, isomalabaricane triterpenoids are present exclusively in specimens of *R. globostellata*. Considering similar morphology and molecular analysis data of the found isomalabaricane-containing *R. globostellata* specimens, and the chemotypes revealed in the present study, further investigations on key enzymes involved in triterpenoid biosynthesis are needed to uncover the biogenesis of isomalabaricane metabolites in these sponges. Finally, an effect of a highly specific symbiont on the isomalabaricane production cannot be ruled out as well, and, therefore, studies on the symbiotic community of the *R. globostellata* sponge using DNA metabarcoding are also expected.

## 4. Materials and Methods

### 4.1. General Experimental Procedures

Optical rotations were measured on a Perkin-Elmer 343 digital polarimeter (Perkin Elmer, Waltham, MA, USA). ^1^H NMR (700.13 MHz) and ^13^C NMR (176.04 MHz) spectra were recorded in CDCl_3_ on a Bruker Avance III 700 spectrometer (Bruker BioSpin, Bremen, Germany). The ^1^H and ^13^C NMR chemical shifts were referenced to the solvent peaks at *δ*_H_ 7.26 and *δ*_C_ 77.0 for CDCl_3_. HRESIMS analyses were performed using a Bruker Impact II Q-TOF mass spectrometer (Bruker, Bremen, Germany). The operating parameters for ESI were as follows: a capillary voltage of 3.5 kV, nebulization with nitrogen at 0.8 bar, and a dry gas flow rate of 7 L/min at a temperature of 200 °C. The mass spectra were recorded within an *m*/*z* mass range of 100–1500. The instrument was operated using the otofControl (ver. 4.1, Bruker Daltonics, Bremen, Germany) and data were analyzed using the DataAnalysis Software (ver. 4.4, Bruker Daltonics, Bremen, Germany). Column chromatography was performed on Sephadex LH-20 (25–100 µm, Pharmacia Fine Chemicals AB, Uppsala, Sweden) and silica gel (KSK, 50−160 mesh, Sorbfil, Krasnodar, Russia). HPLC was carried out using an Agilent 1260 Infinity II chromatograph equipped with a differential refractometer (Agilent Technologies, Santa Clara, CA, USA). Reversed-phase columns YMC-Pack ODS-A (YMC Co., Ishikawa, Japan, 10 × 250 mm, 5 µm and 4.6 mm × 250 mm, 5 µm) and Discovery HS F5-5 (SUPELCO Analytical, Bellfonte, PA, USA, 10 × 250 mm, 5 µm) were used for HPLC. Sorbfil Si gel plates (4.5 × 6.0 cm, 5–17 µm, Sorbpolimer, Krasnodar, Russia) were used for thin-layer chromatography (TLC).

### 4.2. Animal Material

Screened sponge samples were collected either by scuba divers or by dragging during the marine expeditions aboard the R/V “Akademik Oparin” in the Vietnam waters of the South China Sea (Table 1 and Figure 1). Two specimens of *R. globostellata* PIBOC O63-090 (1.0 kg) and PIBOC O63-136 (1.1 kg) were frozen shortly after collection in May 2021 and kept frozen until laboratory analyses. Other specimens were kept in 96% EtOH. The voucher specimens of sponges *R. globostellata* (PIBOC O34-077, PIBOC O49-009, PIBOC O49-030, PIBOC O49-055, PIBOC O63-090, PIBOC O63-136, PIBOC O66-089, PIBOC O66-092, PIBOC O66-109) and *Geodia* sp. (PIBOC O66-120) were deposited at the Collection of marine invertebrates of the G.B. Elyakov Pacific Institute of Bioorganic Chemistry FEB RAS (PIBOC FEB RAS), Vladivostok, Russia. Also, fragments of five specimens used in the molecular analysis were deposited in the Museum (MIMB) of the A.V. Zhirmunsky National Scientific Center of Marine Biology FEB RAS (NSCMB FEB RAS), Vladivostok, Russia. The accession numbers of the deposited MIMB specimens are provided in Table 1.

### 4.3. Morphological Studies

All the studied sponge specimens were fixed in 96% ethanol. The section preparations and the optical and SEM mounts of spicules were prepared using standard methods as described by Hooper [63]. Spicules were counted and measured and photographs of perpendicular sections through the ectosome and choanosome were taken using a ZEISS Primostar 3 optical microscope (Carl Zeiss Microscopy Deutschland GmbH, Oberkochen, Germany). SEM images of spicules were taken using a Zeiss Sigma 300 VP scanning electron microscope (Carl Zeiss Microscopy GmbH, Oberkochen, Germany). Measurements were expressed as “minimum–**mean**–maximum”. The number of measured spicules of each category was 25 for *R. globostellata* and 10 for *Geodia* sp. All spicule dimensions are given in micrometers (μm). The classification system used in this study is in accordance with the World Porifera Database (WPD) [16]; the spicular and morphological nomenclature is according to Łukowiak et al. [64].

### 4.4. Molecular Analysis

DNA was isolated from fragments of five specimens (Table 1) fixed in 96% EtOH using a PureLink™ Genomic DNA Mini Kit (Invitrogen™, Thermo Fisher Scientific, Missouri, TX, USA) according to the manufacturer’s instructions. The primers used for the amplification of 18S and 28S rRNA and ITS1–5.8S–ITS2 gene fragments are listed in Table S4 [65,66,67].

The amplification reaction was carried out in 10 μL of a mixture consisting of DreamTaq polymerase (Thermo Fisher Scientific, Waltham, MA, USA) (0.1 U/μL), 1× Taq Turbo buffer (Evrogen, Moskow, Russia), and forward and reverse primers at a concentration of 0.25 μM each and 0.2 mM dNTPs each in the a Verity thermocycler (Applied Biosystems, Thermo Fisher Scientific, Singapore). Thermocycling conditions were as follows: an initial denaturation step of 3 min at 95 °C, followed by 35 cycles of denaturation at 95 °C for 20 s, annealing at the temperature presented in Table S4 for 30 s, and an extension of 72 °C for 1 min 30 s. This was followed by a final extension step of 72 °C for 7 min and holding at 4 °C. The quality and quantity of PCR products were checked by electrophoresis in 1.5% agarose gel (Gerbu Biotechnik GmbH, Wieblingen, Germany). Amplified DNA fragments were purified with ExoSap-IT (Thermo Fisher Scientific, Waltham, MA, USA) under the conditions recommended by the manufacturer. Amplicons were used as a template for the sequencing reaction using a BigDye Terminator v3.1 Cycle Sequencing Kit (Applied Biosystems, Foster City, CA, USA). The same primers were used for the sequencing reaction as for the amplification. Sequence reaction products were purified from unreacted terminators on Sephadex G-50 columns (GE Healthcare, Vienna, Austria). Dried samples were dissolved in formamide and subjected to electrophoresis in a GA-3500 Genetic Analyzer (Applied Biosystems, Waltham, MA, USA). Sequences were assembled using the Geneious R11 program (ver. 11.1.5, https://www.geneious.com/) and deposited in the NCBI/GenBank database (https://www.ncbi.nlm.nih.gov/nuccore/PV055962 (accessed on 8 February 2025); https://www.ncbi.nlm.nih.gov/nuccore/PV061048 (accessed on 9 February 2025); https://www.ncbi.nlm.nih.gov/nuccore/PV061049 (accessed on 9 February 2025); https://www.ncbi.nlm.nih.gov/nuccore/PV061050 (accessed on 9 February 2025); https://www.ncbi.nlm.nih.gov/nuccore/PV061051 (accessed on 9 February 2025); https://www.ncbi.nlm.nih.gov/nuccore/PV061052 (accessed on 9 February 2025); https://www.ncbi.nlm.nih.gov/nuccore/PV061053 (accessed on 9 February 2025)) under the accession numbers given in Table 1. Taxonomic identification of sponge samples was carried out by comparing the sequences with those available in NCBI/GenBank using the BLASTn algorithm (ver. 2.15.0).

### 4.5. Extraction and Isolation

The *R. globostellata* specimen (PIBOC O63-136, 1.1 kg) was defrosted, sliced, and extracted with EtOH (1.3 L× 3). Concentrated extract (46.4 g) was partitioned between water (300 mL) and EtOAc (300 mL × 3). Concentrated organic layers (7.9 g) were subdivided on Sephadex LH-20 column (2 × 100 cm, CHCl_3_/EtOH, 1:1, loaded in four portions) followed by stepwise gradient silica gel separation (3.5 × 20 cm, CHCl_3_→EtOH, loaded in five portions). As a result, fifteen fractions A–O of increasing polarity were obtained. Yellow fraction J (792.1 mg, eluted with CHCl_3_/EtOH, 5:1) was subjected to reversed-phase HPLC (YMC-Pack ODS-A) in 95% MeOH. Further HPLC purification (Discovery HS F5-5) of collected subfractions led to the isolation of rhabdastrellic acid A (**6**) (17.3 mg, 80% MeOH), stellettins E (**7**) (8.8 mg, 80% MeOH), H (**8**) (1.5 mg, 90% MeOH), and I (**9**) (2.2 mg, 90% MeOH). 17*Z*-rhabdastrellic acid A (**1**, 1.5 mg) was isolated as a minor component among compounds **6** and **7** by further re-chromatography (Discovery HS F5-5) in 80% MeOH. The major yellow fraction E (3.3 g, eluted with CHCl_3_/EtOH, 50:1) was subdivided using stepwise gradient reversed-phase column chromatography (3 × 19 cm, YMC-Pack ODS-A, 10% EtOH→100% EtOH). Then, a half portion of the subfraction eluted with 80% EtOH (yellow, 1.8 g) was purified by silica gel column chromatography (1.5 × 6 cm, CHCl_3_/EtOH, 50:1, loaded in three portions) followed by HPLC (YMC-Pack ODS-A, 95% MeOH) to yield stellettins A (**2**, 9.1 mg), B (**3**, 5.8 mg), C (**4**, 1.0 mg), and D (**5**, 1.0 mg). Finally, the polar fraction M (91.2 mg, eluted with CHCl_3_/EtOH, 1:1) was divided by subsequent HPLC (YMC-Pack ODS-A) in 70% EtOH and re-chromatography in 60% EtOH to yield 2*E*,4*E*-2-methyl-6-oxo-2,4-heptadienoic acid (**10**) (1.0 mg).

Amber glassware and aluminum foil protection were used during the experimental procedures to avoid sunlight-induced interconversion of the corresponding *Z*/*E*-isomers.

### 4.6. Compound Characteristics

17*Z*-rhabdastrellic acid A (**1**): Yellowish oil; [*α*]_D_^20^–93.0 (*c* 0.1, CHCl_3_); ^1^H and ^13^C NMR data (CDCl_3_), Table 1; (–)HRESIMS *m*/*z* 463.2850 [M–H]^−^ (calcd for C_30_H_39_O_4_ 463.2854); and (–)HRESIMS/MS of the ion [M–H]^−^ at *m*/*z* 463.2837: 419.2943 [M–H–CO_2_]^−^.

### 4.7. LC-MS Procedures

Purification and desalting of the ethanol extracts were performed by solid-phase extraction using an Oasis HLB extraction cartridge (60 mg, Waters, Medford, MA, USA). The sorbent was conditioned with 3 mL of methanol followed by 3 mL of water. The filtered extract (250 μL) was loaded onto the cartridge, followed by a wash with 1 mL of water. Metabolites were eluted with 1 mL of 75% acetonitrile in water containing 0.1% formic acid. After drying, the samples were reconstituted in 200 µL of 50% acetonitrile in water (*v*/*v*) and subjected to LC-MS analyses. Previously isolated isomalabaricane triterpenoids used as authentic standards were dissolved in 50% acetonitrile in water (*v*/*v*) at a concentration of 0.2 mg/mL and subjected to LC-MS analysis.

The analysis was carried out using a Bruker Elute UHPLC system (Bruker Daltonics, Bremen, Germany) consisting of an Elute Pump HPG 1300, an Elute Autosampler UHPLC, and an Elute Column Oven coupled to a Bruker Impact II Q-TOF mass spectrometer (Bruker Daltonics, Bremen, Germany) equipped with an electrospray ionization (ESI) source. Chromatographic separation was performed on an InfinityLab Poroshell 120 SB-C18 column (2.1 × 150 mm, 1.9 μm, Agilent Technologies, Santa Clara, CA, USA). The mobile phases were 0.1% formic acid in water (eluent A) and 0.1% formic acid in acetonitrile (eluent B). The gradient was programmed as follows: 5–25% B (0–2 min), 25–80% B (2–19 min), 80–100% B (19–20 min), isocratic at 100% B (20–25 min), then reduced to 5% B (25.0–25.2 min). The column was re-equilibrated for 3.8 min before the next injection. The flow rate was 0.4 mL/min, the column temperature was 45 °C, and the injection volume was 4 μL in the partial loop injection mode.

Mass spectrometry detection was performed in negative ion mode ESI. Optimized ionization parameters were set as follows: a capillary voltage of 4 kV, nebulization with nitrogen at 2.0 bar, and a dry gas flow rate of 8 L/min at 210 °C. Mass spectra were acquired in the *m*/*z* range of 80–1700 at 2 Hz. Collision-induced dissociation (CID) product-ion spectra were recorded in the autoMS/MS mode, selecting the top 2 features of each scan, with a sampling rate of 3 Hz and an isolation width of 4 Th. Collision energies for MS/MS experiments were set as follows: *m*/*z* 100, 15 eV; *m*/*z* 200, 35 eV; *m*/*z* 400, 50 eV; *m*/*z* 800, 95 eV; and *m*/*z* 1000, 120 eV. The stepping option for collision energy was applied, acquiring 50% of each MS/MS scan at 60% of the set collision energy, in order to obtain the most informative fragmentation spectra. The instrument was calibrated using the ESI-L Low Concentration Tuning Mix (Agilent Technologies, Santa Clara, CA, USA). The mass spectrometer was operated using otofControl (ver. 4.1, Bruker Daltonics, Bremen, Germany). The DataAnalysis software (ver. 4.3, Bruker Daltonics, Bremen, Germany) was used for analyzing the MS data and for generation of molecular formulas using the SmartFormula module with a 10 ppm error window.

The LC-MS raw files were converted to an open-source mzML format using the MSConvert [68]. The mzML files were then processed, using MZmine 2.53 [69], for feature detection, identification, and generation of an .mgf file containing the MS/MS data of all detected features. For identification of known isomalabaricanes, data was searched against an in-house spectral library in .mgf format, which contained MS/MS spectra and retention times of 16 previously isolated isomalabaricanes. Detailed MZmine parameters are provided in the Supplementary Material (Table S5). The combined .mgf file was then used as input for molecular networking analysis. A molecular network was created using the Feature-Based Molecular Networking (FBMN) [70] workflow (version release_28.2) on the GNPS platform [43] with a parent mass tolerance of 0.01 Da and a fragment-ion tolerance of 0.02 Da. A molecular network was created with edges filtered to have a cosine score above 0.7 and more than four matching peaks. Afterwards, the edges between two nodes were kept in the network if, and only if, each of the nodes appeared in each other’s respective top 10 most similar nodes. The maximum molecular family size was set to 100, and the lowest-scoring edges were sequentially removed until the family size was below this threshold. The resulting networks were visualized using Cytoscape (ver. 3.9.1) [71]. The molecular networks can be accessed at [72]. Principal component analysis and hierarchical cluster analysis were performed using the MetaboAnalyst 6.0 platform [73]. Prior to analysis, the data were sum-normalized, log-transformed, and auto-scaled.

## 5. Conclusions

In the present study, we have combined wide screening of the isomalabaricane sources among sponges from the South China Sea, reliable taxonomic identification of the found samples, isolation and structure elucidation of the individual target metabolites, and their application for the in-depth analysis of the isomalabaricane chemical space using a modern metabolomic approach. The new experimental data have confirmed that among marine sponges, inhabiting Vietnamese waters, isomalabaricane triterpenoids are present exclusively in the *Rhabdastrella globostellata* specimens. Nine individual isomalabaricane triterpenoids have been isolated, including new 17*Z*-rhabdastrellic acid A. Annotation of the data obtained by LC-MS profiling and MS/MS-molecular networking has revealed clear divergence in the biosynthesis of these triterpenoids in the studied sponges depending on the collection site. Thus, for the Vietnamese specimens of *R. globostellata*, two primary chemotypes have been discovered and characterized with the individual sets of annotated compounds. Moreover, series of new extremely rare nitrogen-containing isomalabaricanes and glycosides of this structural class have been detected by applying a combination of MS and MS/MS techniques.

## Figures and Tables

**Table 1:** List of the species and sampling locality data.

Sample	Voucher ^1^	Month, Year	Locality	Collection Coordinates	18S rRNA 28S rRNA (Y/N) ^2^	Identification
1	PIBOC O34-077	May, 2007	Bath Long Vi Island	20°09.326′ N 107°44.357′ E	**N**	*R. globostellata*
2	PIBOC O49-009	November, 2017	Con Co Island	17°09.67′ N 107°19.8′ E	**N**	*R. globostellata*
3	PIBOC O49-030	November, 2017	Con Co Island	17°08.67′ N 107°20.56′ E	**N**	*R. globostellata*
4	PIBOC O49-055	November, 2017	Con Co Island	17°10.03′ N 107°20.33′ E	**N**	*R. globostellata*
5	PIBOC O63-090 (MIMB 50534)	May, 2021	Con Co Island	17°09.4′ N 107°20.0′ E	**Y** (PV061048)	*R. globostellata*
6	PIBOC O63-136 (MIMB 50532)	May, 2021	Hon Gio	17°54.7′ N 106°40.2′ E	**Y** (PV055962, PV061049, PV061050)	*R. globostellata*
7	PIBOC O66-089 (MIMB 50535)	May, 2023	Phan Thiet	10°39.3′ N 108°41.5′ E	**Y** (PV061051)	*R. globostellata*
8	PIBOC O66-092 (MIMB 50533)	May, 2023	Phan Thiet	10°39.3′ N 108°41.5′ E	**Y** (PV061052)	*R. globostellata*
9	PIBOC O66-109 (MIMB 50536)	May, 2023	Phan Thiet	10°38.5′ N 108°42.1′ E	**Y** (PV061053)	*R. globostellata*
10	PIBOC O66-120	May, 2023	Phan Thiet	10°38.5′ N 108°42.1′ E	**N**	*Geodia* sp.

^1^ Fragments of five specimens used in the molecular analysis were deposited in the museum of NSCMB FEB RAS. The accession numbers of the corresponding specimens with the acronym MIMB are provided in parentheses. ^2^ Sequences were deposited in the NCBI/GenBank database under the accession numbers given in parentheses.

**Table 2:** ^1^H and ^13^C NMR data of compound **1** in CDCl_3_.

No. ^1^	1 (700 and 176 MHz)	
	*δ*_H_ Mult (*J* in Hz)	*δ*_C_
1*α* 1*β*	2.17, m 1.52, m	31.4
2*α* 2*β*	2.72, ddd (16.8, 11.9, 5.8) 2.38, m	33.5
3		219.0
4		46.9
5*α*	2.41, m	45.5
6*α* 6*β*	1.62, m 1.54, m	19.8
7a 7b	2.24, m 2.18, m	38.5
8		45.0
9*β*	1.88, dd (12.9, 9.4)	47.9
10		34.8
11	2.23, m (2H)	36.7
12		206.9
13		146.5
14		141.7
15	6.66, d (14.9)	133.6
16	7.19, dd (14.9, 11.6)	130.4
17	6.31, d (11.6)	133.8
18	2.37, s	14.6
19	0.86, s	23.5
20		136.8
21	2.06, s	20.6
22	7.21, d (15.0)	135.9
23	6.63, dd (15.0, 11.6)	125.8
24	7.45, d (11.6)	140.4
25		126.9
26		171.5
27	2.03, s	12.7
28	1.12, s	29.2
29	1.06, s	19.4
30	1.43, s	25.9

^1^ Assignments were made based on of HSQC, HMBC, and ROESY data.

**Table 3:** Retention times and molecular masses of standard isomalabaricanes and their relative contents in the ethanolic extracts of *R. globostellata* specimens based on the LC–ESI MS analysis.

Compound ^1^ [Ref.]	Molecular Formula	RT, min	[M–H]^−^ Meas	Relative Content, % ^2^								
				PIBOC O34-077	PIBOC O49-009	PIBOC O49-030	PIBOC O49-055	PIBOC O63-090	PIBOC O63-136	PIBOC O66-089	PIBOC O66-092	PIBOC O66-109
Rhabdastrelloside A [13]	C_44_H_73_NO_14_	9.53	838.4961	3.6	51.4	87.1	53.5	14.1	100.0	2.1	8.7	3.5
Rhabdastrelloside B [13]	C_44_H_73_NO_13_	10.54	822.5014	1.3	69.6	100	51.0	17.7	29.5	1.0	4.6	4.4
Stellettin W [14]	C_22_H_32_O_6_	10.93	391.2135	60.5	89.1	26.8	17.8	100.0	61.2	23.0	24.0	37.0
Globostelletin N [12,41]	C_30_H_40_O_5_	11.07	479.2805	41.7	28.9	14.7	20.9	37.7	12.6	99.3	91.0	100.0
Stellettin S [12]	C_19_H_28_O_3_	11.41	303.1968	67.9	73.6	42.8	51.3	100.0	40.2	0	0	0.3
Globostelletin M [12,41]	C_30_H_40_O_5_	12.11	479.2801	100.0	66.4	19.5	38.1	52.2	17.2	8.6	9.4	9.1
Globostelletin K [12,41]	C_30_H_40_O_5_	12.28	479.2792	84.3	82.8	25.6	60.5	100.0	70.5	1.5	2.8	5.0
Jaspolide F [5,42]	C_25_H_34_O_4_	13.09	397.2374	100.0	78.6	78.5	51.0	43.5	20.9	0.3	0.5	0.6
Globostelletin G [4]	C_25_H_34_O_4_	13.50	397.2372	100.0	78.6	60.5	44.5	48.4	13.6	0	0	0
RAA (**6**) [33]	C_30_H_40_O_4_	16.87	463.2857	90.1	36.8	100.0	83.6	47.3	88.5	1.1	0.6	1.6
17Z-RAA (**1**)	C_30_H_40_O_4_	17.27	463.2858	35.6	12.2	32.4	42.1	100.0	43.9	0.1	0.2	0.1
Stellettin E (**7**) [34]	C_30_H_40_O_4_	17.37	463.2852	78.2	17.2	66.3	74.5	100.0	68.4	0.4	0	0.1
Stellettin A (**2**) [6]	C_30_H_38_O_4_	17.54	461.2688	32.0	24.1	66.6	86.0	83.8	100.0	0.8	0	2.9
Stellettin B (**3**) [6]	C_30_H_38_O_4_	17.61	461.2698	14.4	8.7	30.7	93.0	83.1	100.0	0.5	0	2.2
Stellettin H (**8**) [37]	C_32_H_44_O_5_	20.03	507.3111	32.6	14.2	27.9	56.6	100.0	48.0	1.4	0.4	2.0
Stellettin I (**9**) [37]	C_32_H_44_O_5_	20.25	507.3112	100.0	11.5	19.5	56.1	99.7	58.8	0	0	0.7

^1^ Structures of isomalabaricanes used as standards for LC–ESI MS analyses are given in Figures S16 and S18. ^2^ Normalized to compound maximum across samples.

## Data Availability

Data are contained within the article and Supplementary Materials.

## Supplementary Materials

The following supporting information can be downloaded at: https://www.mdpi.com/article/10.3390/md23120466/s1, Figures S1–S3: Images, descriptions, and some details for the studied Vietnamese sponges [5,11,12,13,14,27]; Figure S4: (−)HRESIMS data of 17*Z*-rhabdastrellic acid A (**1**); Figures S5−S14: NMR spectra of isolated isomalabaricanes in CDCl_3_; Figure S15: A representative LC–ESI MS chromatograms in negative ion mode for *R*. *globostellata* samples; Figure S16: Structures of isomalabaricanes used as standards for LC–ESI MS analyses; Figure S17: Feature-based molecular network from LC-ESI MS/MS dataset of nine *Rhabdastrella* extracts; Figure S18: MS/MS spectra of [M–H]^−^ ions of isomalabaricane standards; Table S1: Sizes of spicules of *R*. *globostellata*; Table S2: ^1^H and ^13^C NMR data of stellettins A (**2**) and D (**5**) in CDCl_3_ [36]; Table S3: Isomalabaricanes in the ethanolic extracts of *R*. *globostellata* specimens detected by LC–ESI MS/MS and FBMN analysis; Table S4: Primers used for the amplification of 18S and 28S rRNA, and ITS1–5.8S–ITS2 gene fragments [65,66,67]; Table S5: Batch steps and parameters used for data preprocessing in MZmine.
