# Supplementary material for: Isomalabaricane Chemical Composition of Vietnamese Marine Sponges Inspected by Metabolomic and Chemical Approaches
Source: Mar Drugs. 2025 Dec 5;23(12):466. doi: 10.3390/md23120466 (PMC12735236; doi:10.3390/md23120466)
Supplement: Supplementary file 1 [file marinedrugs-23-00466-s001.zip › marinedrugs-3958054-supplementary.pdf]

Supplementary Materials

Isomalabaricane chemical composition of Vietnamese marine sponges inspected by metabolomic and chemical approaches

Sophia A. Kolesnikova[1*†], Anastasia B. Kozhushnaya[1†], Vladimir A. Shilov[2], Andrey D. Kukhlevsky[2], Anatoly I. Kalinovsky[1], Roman S. Popov[1], Pavel S. Dmitrenok*[1] and Natalia V. Ivanchina[1]

[1] G.B. Elyakov Pacific Institute of Bioorganic Chemistry, Far Eastern Branch, Russian Academy of Sciences, Pr. 100-Let Vladivostoku 159, 690022 Vladivostok, Russia; sovin81@inbox.ru (S.A.K.); kozhushnaia.ab@mail.ru (A.B.K.); kaaniw@piboc.dvo.ru (A.I.K.); popov_rs@piboc.dvo.ru (R.S.P.); paveldmt@piboc.dvo.ru (P.S.D.); ivanchina@piboc.dvo.ru (N.V.I.);

[2] A.V. Zhirmunsky National Scientific Center of Marine Biology, Far Eastern Branch, Russian Academy of Sciences, ul. Palchevskogo 17, 690041 Vladivostok, Russia; shilvl@yandex.ru (V.A.S.); ad.kukhlevskiy@gmail.com(A.D.K.);

* Correspondence: sovin81@inbox.ru (S.A.K.); paveldmt@piboc.dvo.ru (P.S.D.); Tel.: +7-423-231-1168 (P.S.D.);

† These authors contributed equally to this work.

Contents:

S1 Images of Vietnamese sponge samples
S2 The morphology description for the Vietnamese marine sponge *Geodia* sp. PIBOC O66-120
S3 Some features and chemical characteristics for the Vietnamese sponge *Rhabdastrella globostellata* PIBOC O38-301
S4 (−)HRESIMS of 17*Z*-rhabdastrellic acid A (**1**) and MS/MS spectrum of its $[M-H]^-$ precursor ion at *m/z* 463
S5 ^{1}H NMR spectrum of 17*Z*-rhabdastrellic acid A (**1**) in $CDCl_3$ (700 MHz)
S6 ^{13}C NMR spectrum of 17*Z*-rhabdastrellic acid A (**1**) in $CDCl_3$ (176 MHz)
S7 HSQC spectrum of 17*Z*-rhabdastrellic acid A (**1**) in $CDCl_3$ (700 MHz)
S8 HMBC spectrum of 17*Z*-rhabdastrellic acid A (**1**) in $CDCl_3$ (700 MHz)
S9 COSY spectrum of 17*Z*-rhabdastrellic acid A (**1**) in $CDCl_3$ (700 MHz)
S10 ROESY spectrum of 17*Z*-rhabdastrellic acid A (**1**) in $CDCl_3$ (700 MHz)
S11 ^{1}H NMR spectrum of stellettin A (**2**) in $CDCl_3$ (700 MHz)
S12 ^{13}C NMR spectrum of stellettin A (**2**) in $CDCl_3$ (176 MHz)
S13 ^{1}H NMR spectrum of stellettin D (**5**) in $CDCl_3$ (700 MHz)
S14 ^{13}C NMR spectrum of stellettin D (**5**) in $CDCl_3$ (176 MHz)
S15 A representative LC–ESI MS chromatograms in negative ion mode for *R. globostellata* samples
S16 Structures of isomalabaricanes used as standards for LC–ESI MS analyses
S17 Feature-based molecular network from LC-ESI MS/MS dataset of nine *Rhabdastrella* extracts
S18 MS/MS spectra of $[M-H]^-$ ions of isomalabaricane standards

Table S1 Sizes of spicules of *R. globostellata* (Carter, 1883)
Table S2 ^{1}H and ^{13}C NMR data of stellettins A (**2**) and D (**5**) in $CDCl_3$ (700 MHz)
Table S3 Isomalabaricanes in the ethanolic extracts of *R. globostellata* specimens detected by LC–ESI MS/MS and FBMN analysis
Table S4 Primers used for the amplification of 18S and 28S rRNA, and ITS1–5.8S–ITS2 gene fragments
Table S5 Batch steps and parameters used for data preprocessing in MZmine

S1 Images of Vietnamese sponge samples

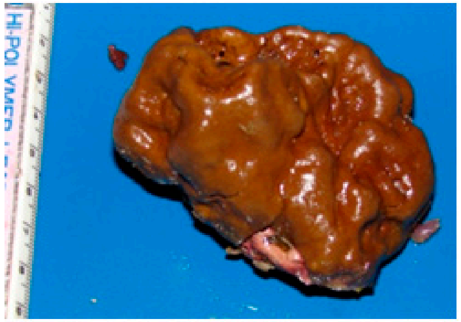

PIBOC 034-077

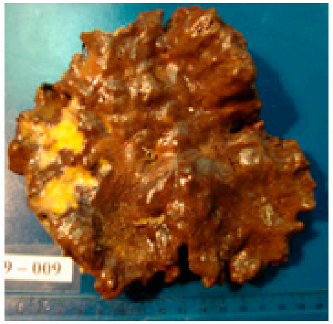

PIBOC 049-009

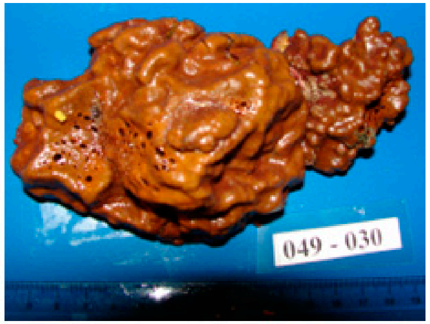

PIBOC 049-030

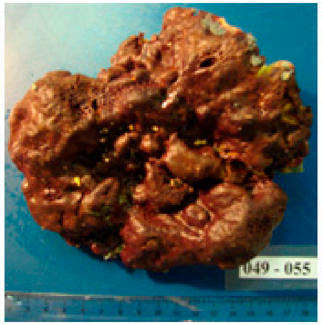

PIBOC 049-055

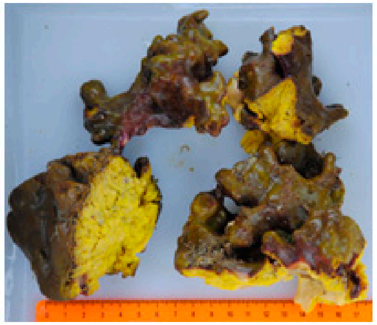

PIBOC 063-090

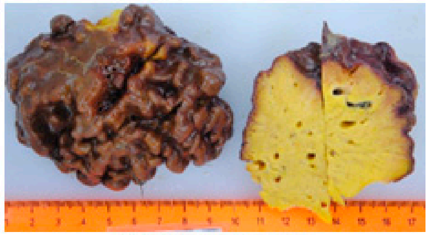

PIBOC 063-136

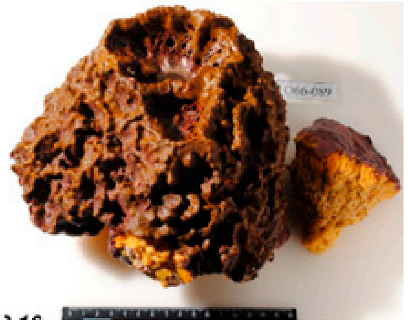

PIBOC 066-089

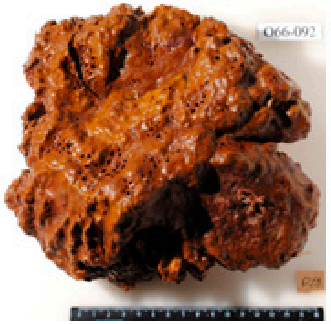

PIBOC 066-092

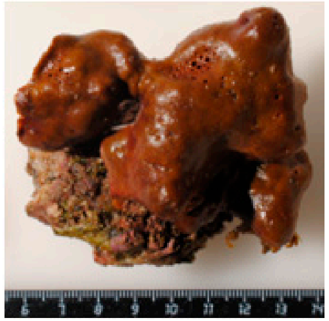

PIBOC 066-109

S2 The morphology description for the Vietnamese marine sponge *Geodia* sp. PIBOC O66-120

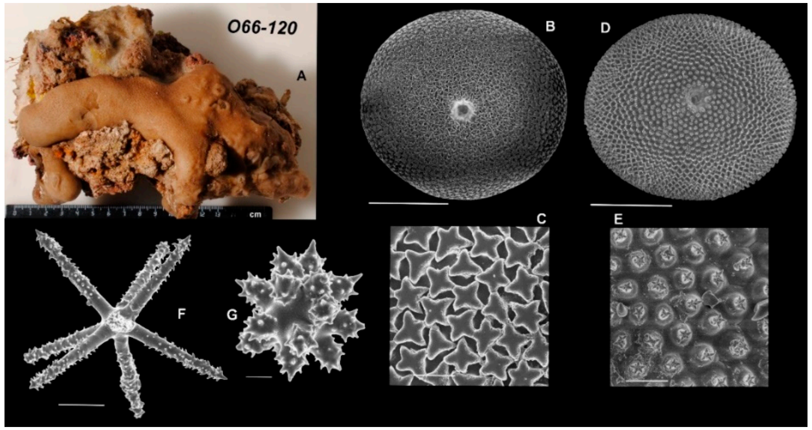

Family **Geodiidae** Gray, 1867

Genus ***Geodia*** Lamarck, 1815

***Geodia* sp.** specimen PIBOC O66-120

Geodia sp., specimen PIBOC O66-120: habitus of a fresh specimen (A), SEM-images of spicules (B–G), sterrasters and surface of warty rosettes (B–E), acanthoxyaster (F), acanthostrongylaster (G). Scale bars: 50 µm for B and D, 5 µm for C, E, F, and 1 µm for G.

An irregularly shaped sponge that forms a thick crust on the surface of coral limestone (A). The surface is rough due to sterrasters and protruding ends of the cortical oxeas. The living sponge is dark beige. The surface and skeleton are typical for the genus: "Cortex conspicuous, formed by a crust of sterrasters with a layer of euasters in the outer zone. Cladome of the triaenes located at the cortex. Megascleres (oxeas and triaenes) radially arranged at the peripheral zone of the sponge, oxeas more disorganized in arrangement in the interior. Inhalant and exhalant orifices arranged in clusters under a sieve of the cortex (cribriporal)." [27].

Spicules (B–G). The authors did not aim to make a complete identification of the specimen, as it was enough to confirm the belonging to the genus *Geodia*. Therefore, a brief description of the set of spicules is given, as well as SEM-images of microsclera. Orthotriaenes: rhabdomes 1658.3–**1876.9**–2010.1; cladomes 427.1–**668.3**–1005; clads 175.9–**324.3**–502.5; oxeas 1532.7–**2035.2**–2386.9 × 23.5–**31.3**–41.1; small oxeas 290.7–**325.8**–340.9 × 13.2–**15.8**–17.7; cortical oxeas 188.5–**200.5**–224.7 × 3.7–**4.4**–4.9; sterrasters (B–E) 117.3–**127.5**–137.9 × 86.2–**106.5**–118.0; acanthoxyasters (F) 18.1–**28.0**–32.3; acanthostrongylasters (G) 3.5–**3.9**–4.4.

27. Uriz, M.J. *Kluwer Academic/Plenum Publ., New York* **2002**.

S3 Some features and chemical characteristics for the Vietnamese sponge *Rhabdastrella globostellata* PIBOC O38-301

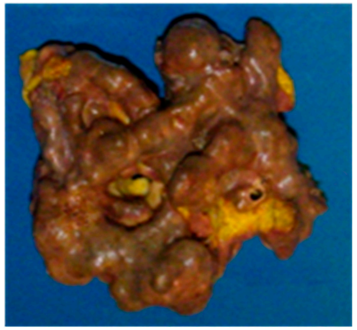

R. globostellata PIBOC O38-301
('brain' morphotype)

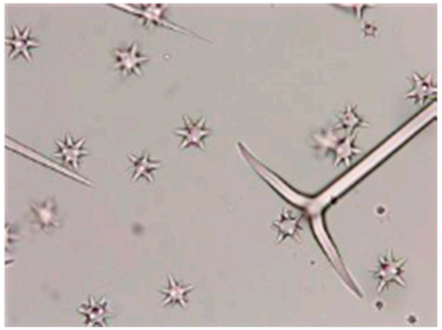

Picture from spicule slide.

Sponge collection: Cham Island, Vietnam, May 2010
(15°54.3'N, 108°31.9'E; 7–12 m).
The specimen was identified by Dr. Paco Cárdenas [11]
(Department of Pharmaceutical Biosciences, Uppsala University, Sweden),

A total of **19 isomalabaricanes were isolated** [5, 12-14], including

triterpenoids: stellettins Q, R, and globostellettins K, M, N;

***nor*-triterpenoids**: cyclobutastellettolides A and B, stellettins S-X, jaspolide F, globostellettins E, F, G;

3-*O*-glycosides: rhabdastrellosides A and B.

TLC for PIBOC O38-301 EtOH extract
in $CHCl_3$-EtOH 20:1
(treated with H_2SO_4 aerosol and heated)

5. Kolesnikova, S.A. et al. *J. Nat. Prod.* **2019**, *82*, 3196–3200. DOI: 10.1021/acs.jnatprod.9b00824.
11. Cárdenas, P. et al. *Mar. Drugs* **2022**, *20*, 190. DOI: 10.3390/md20030190
12. Kolesnikova, S.A. et al. *Molecules* **2021**, *26*, 678. DOI: 10.3390/molecules26030678.
13. Kozhushnaya, A.B. et al. *Mar. Drugs* **2023**, *21*, *554*. DOI: 10.3390/md21110554
14. Kozhushnaya, A.B. et al. *Chem. Nat. Compd.* **2024**, *60*, 1056–1060. DOI: 10.1007/s10600-024-04519-9

S4 (−)HRESIMS of 17*Z*-rhabdastrellic acid A (**1**) and MS/MS spectrum of its [M−H]⁻ precursor ion at *m/z* 463

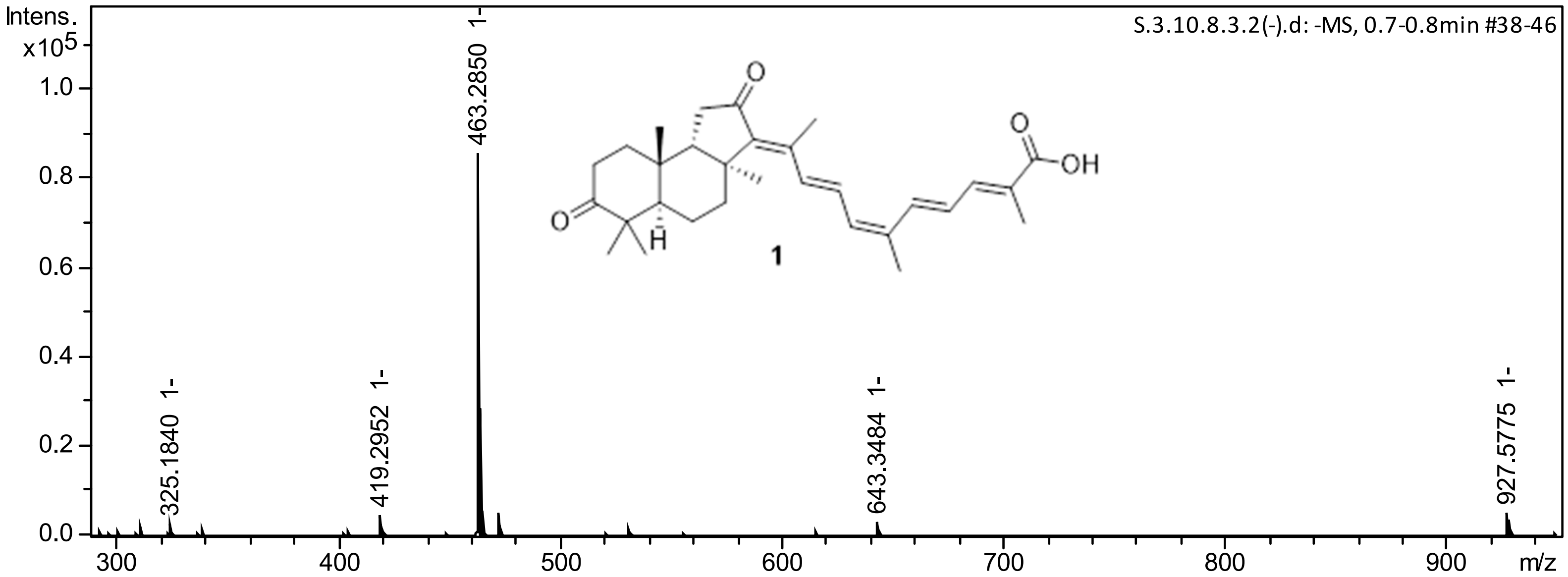

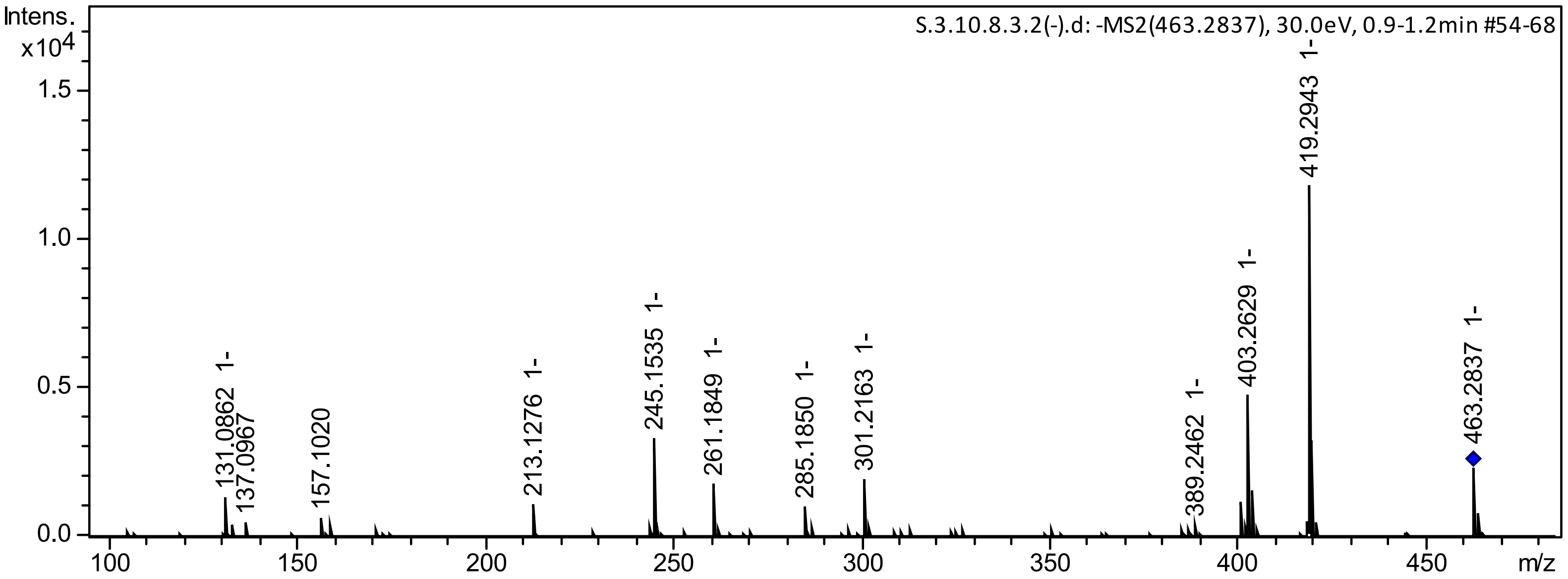

S5 ^{1}H NMR spectrum of 17*Z*-rhabdastrellic acid A (**1**) in $CDCl_3$ (700 MHz)

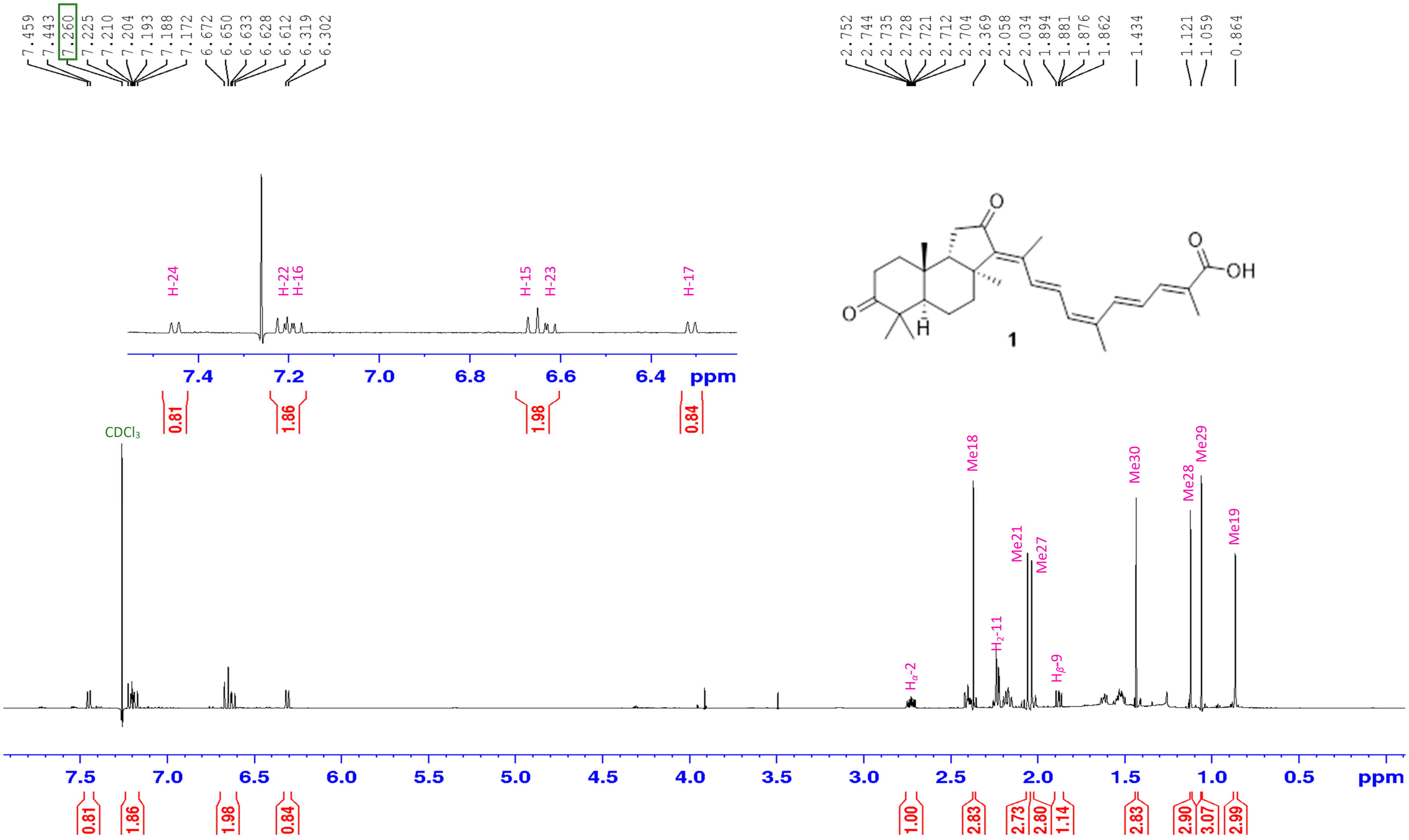

S6 ^{13}C NMR spectrum of 17*Z*-rhabdastrellic acid A (**1**) in $CDCl_3$ (176 MHz)

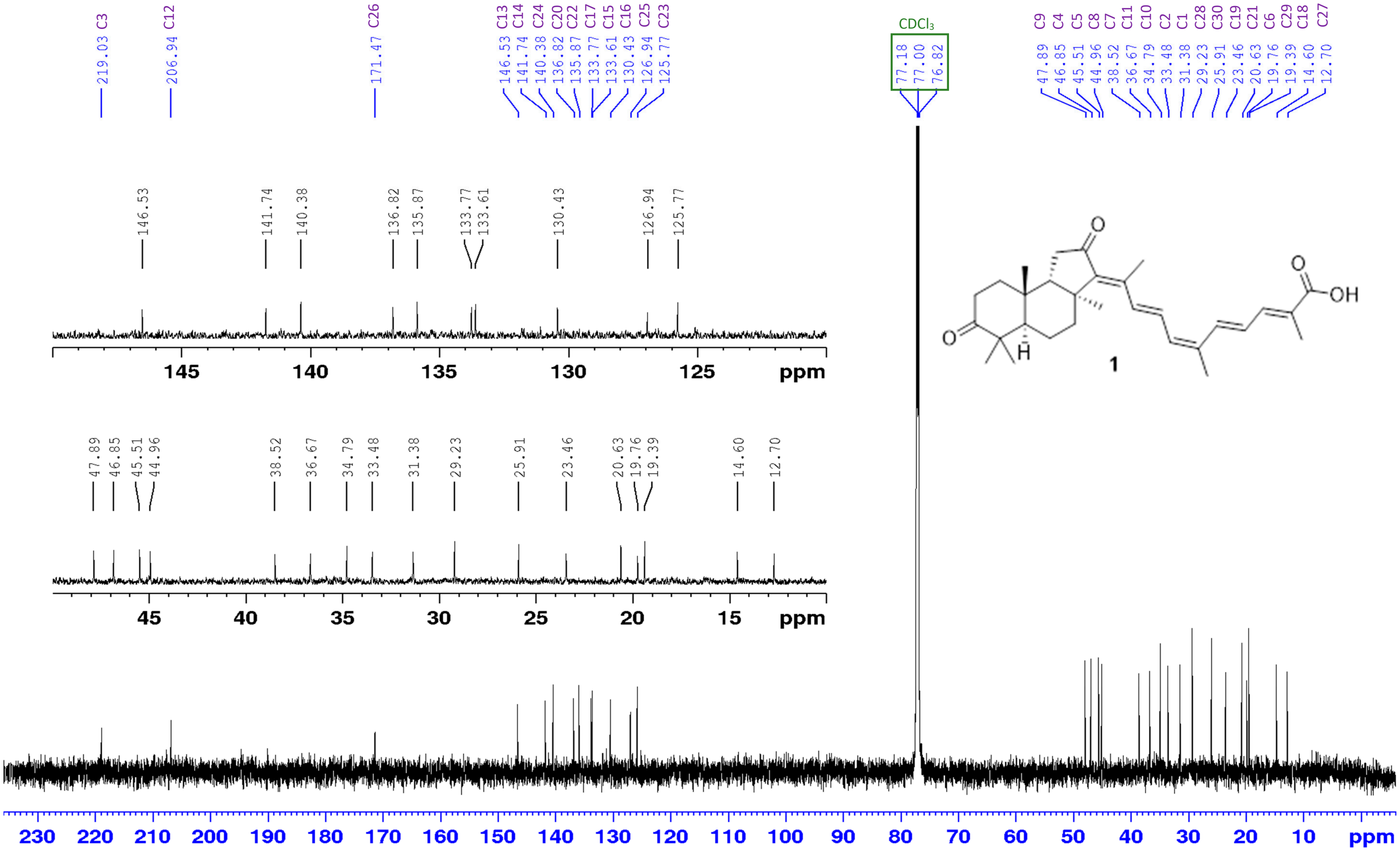

S7 HSQC spectrum of 17*Z*-rhabdastrellic acid A (**1**) in $CDCl_3$ (700 MHz)

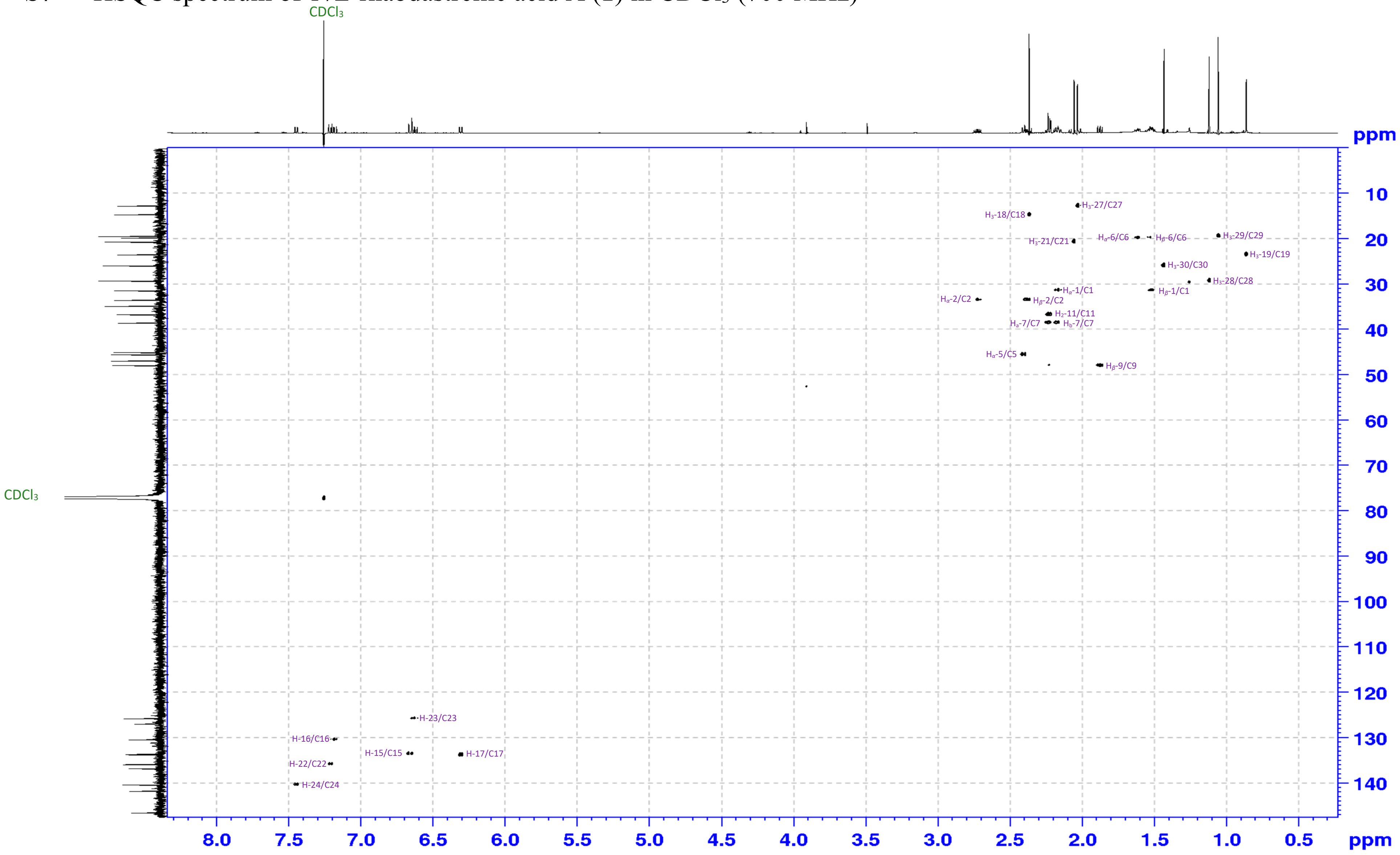

S8 HMBC spectrum of 17*Z*-rhabdastrellic acid A (**1**) in $CDCl_3$ (700 MHz)

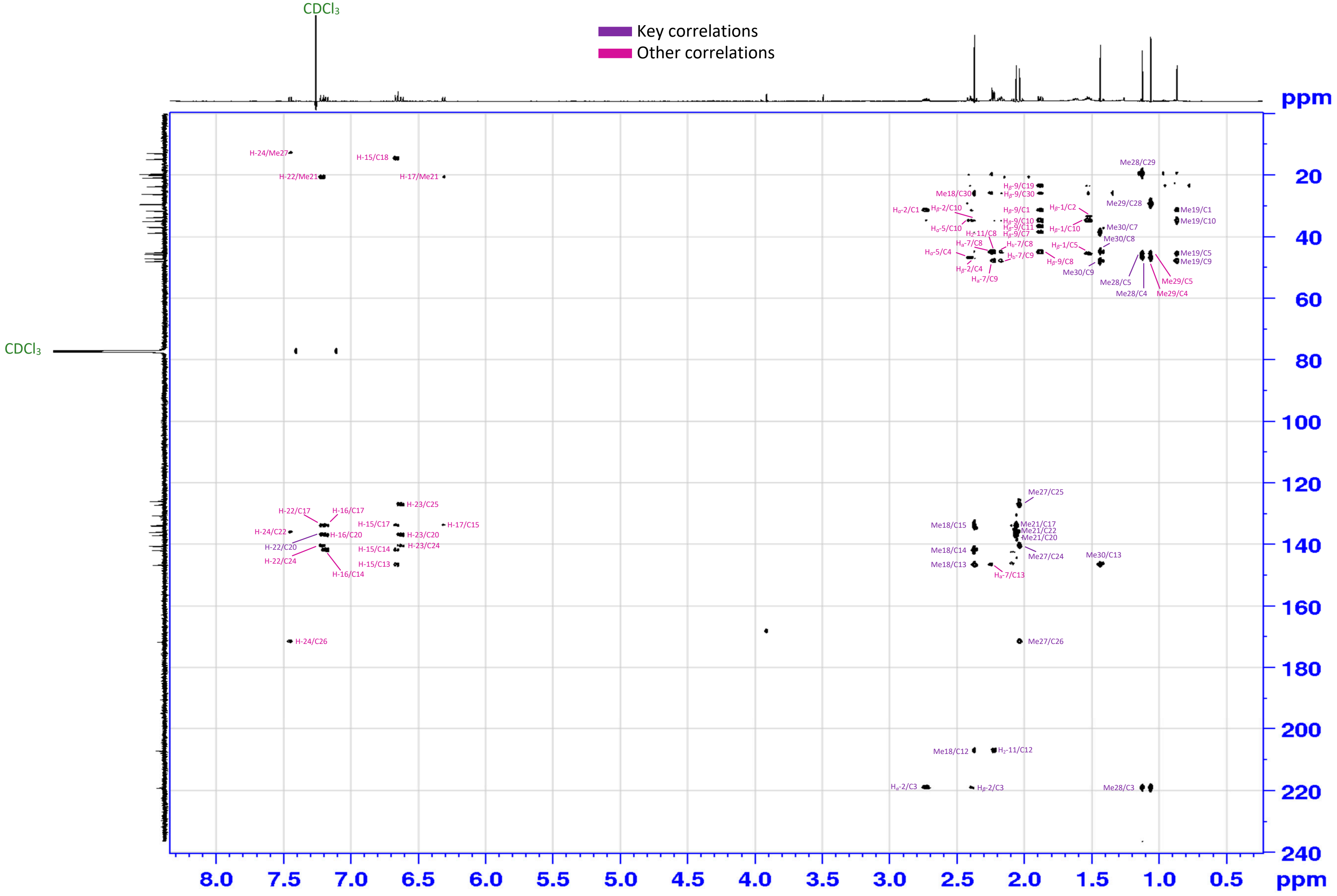

S9 COSY spectrum of 17*Z*-rhabdastrellic acid A (**1**) in $CDCl_3$ (700 MHz)

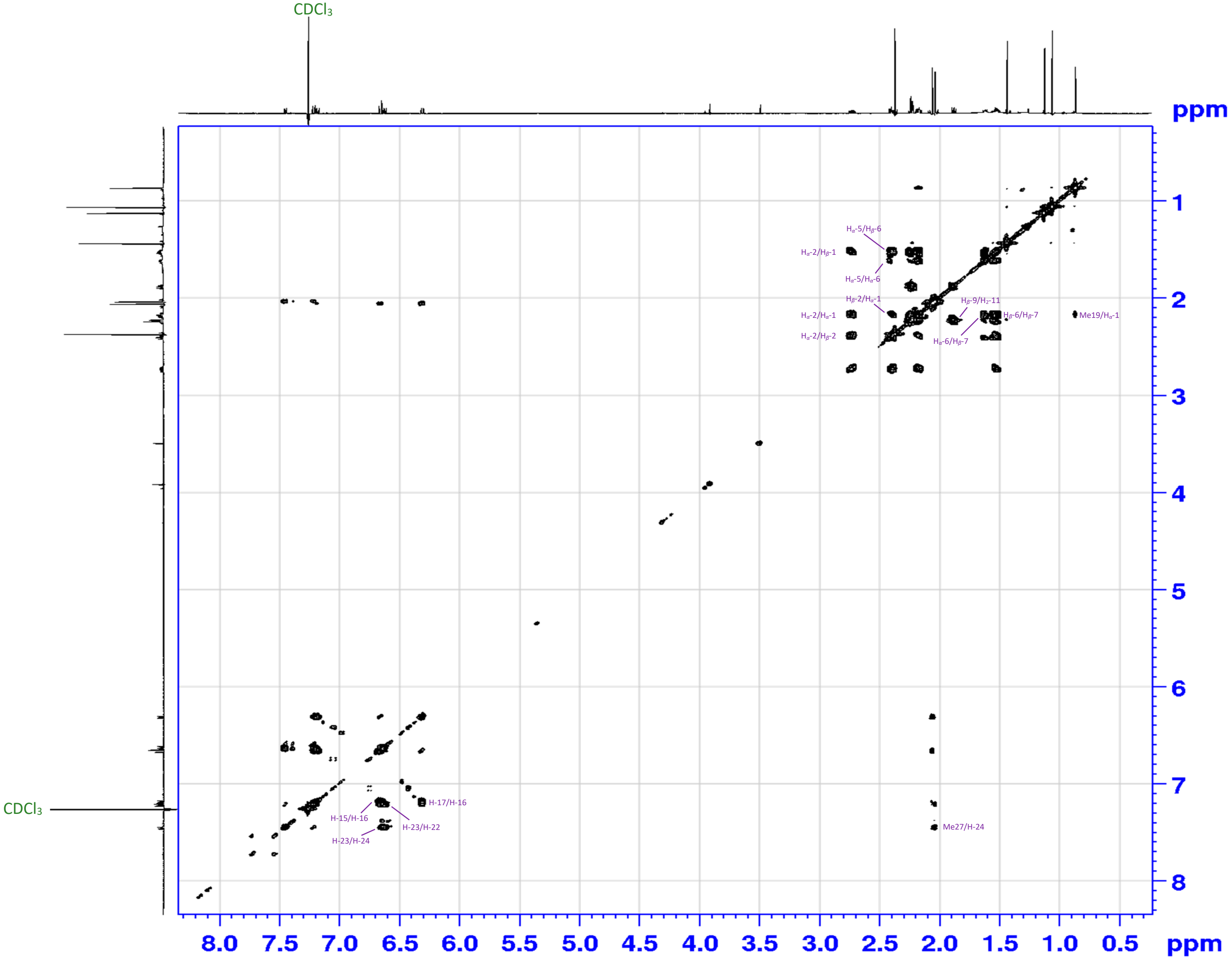

S10 ROESY spectrum of 17*Z*-rhabdastrellic acid A (**1**) in $CDCl_3$ (700 MHz)

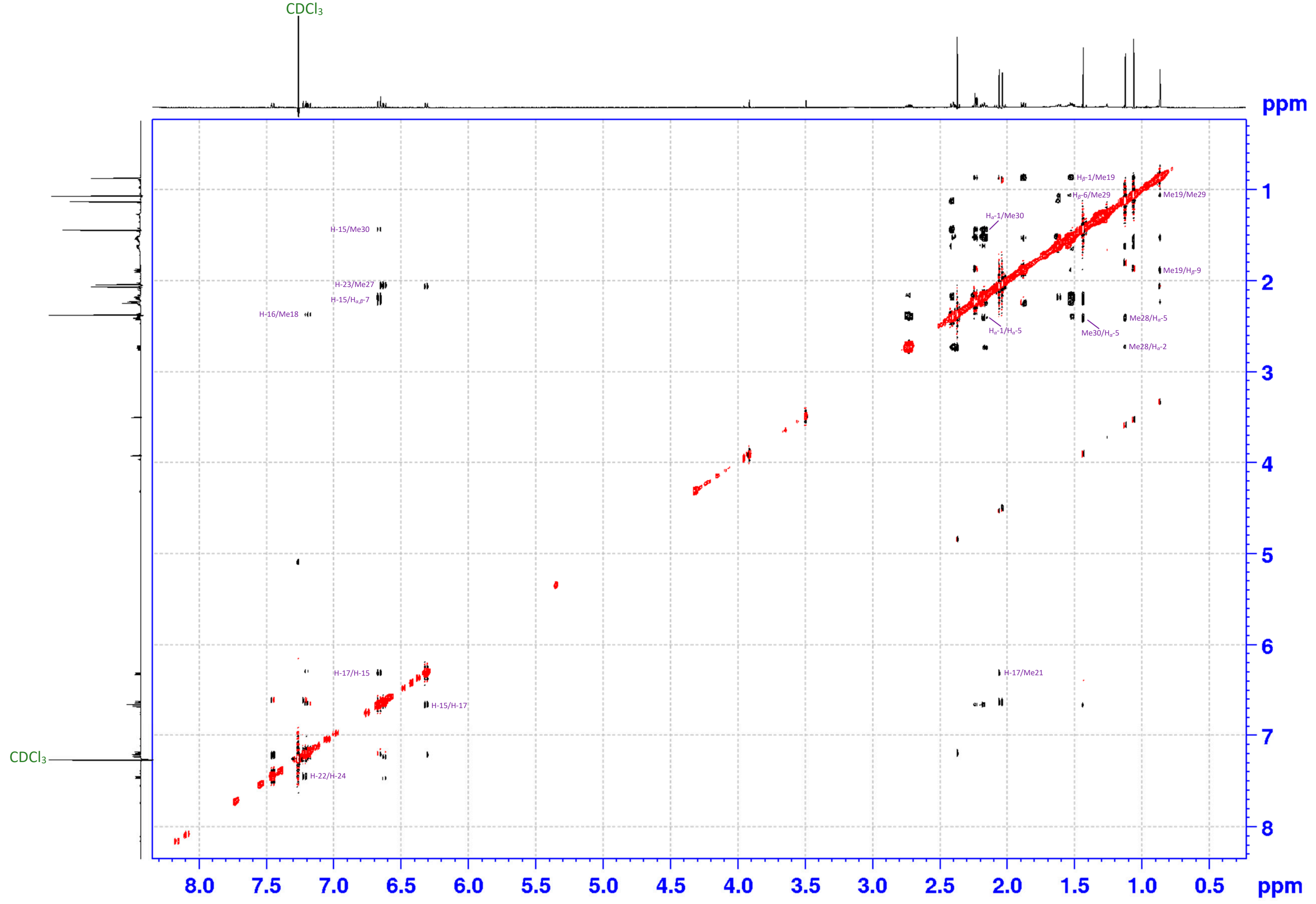

S11 ^{1}H NMR spectrum of stellettin A (**2**) in $CDCl_3$ (700 MHz)

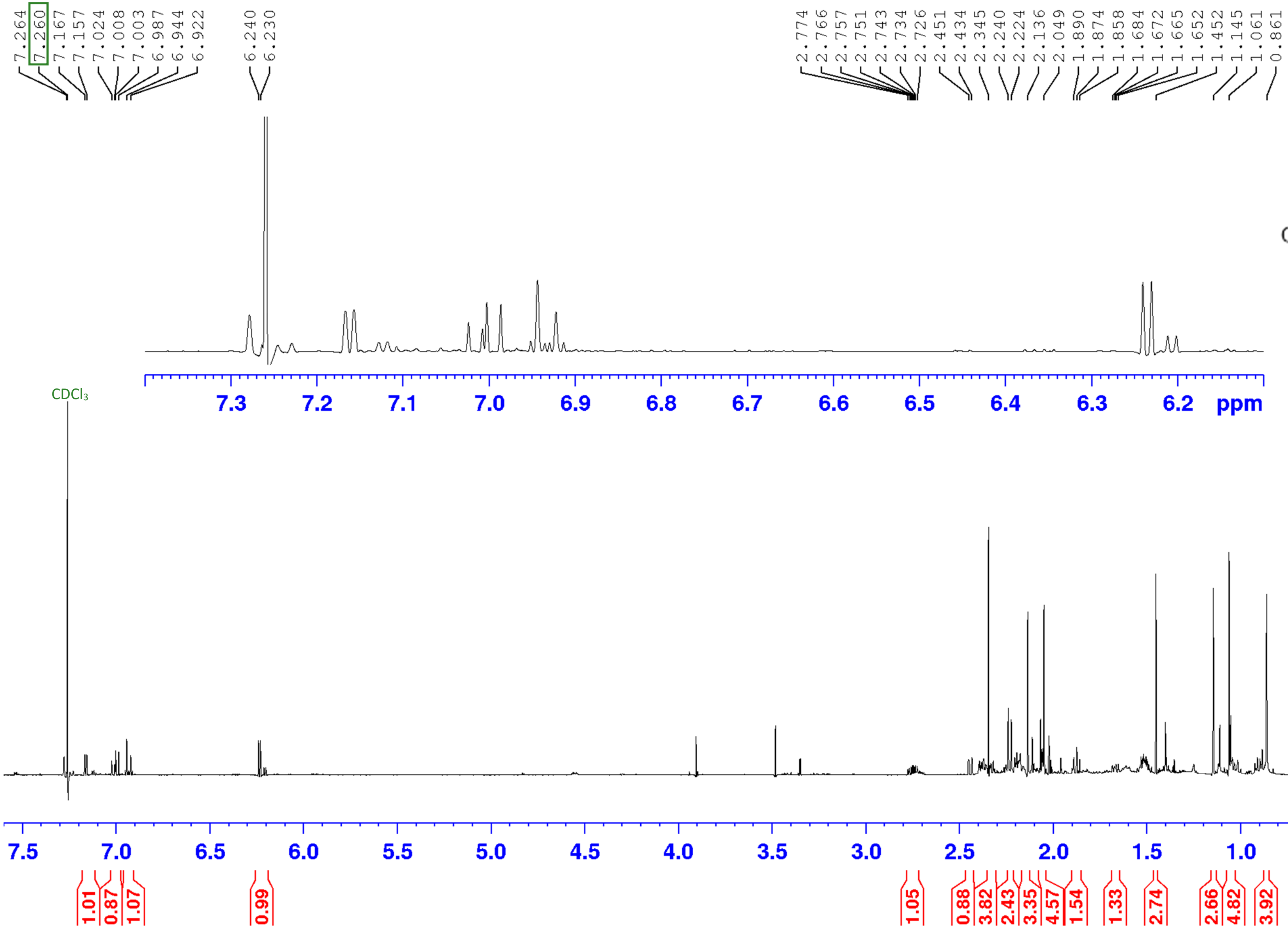

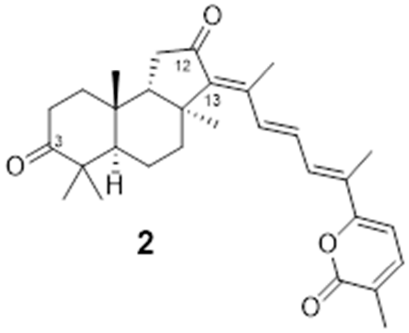

S12 ^{13}C NMR spectrum of stellettin A (**2**) in $CDCl_3$ (176 MHz)

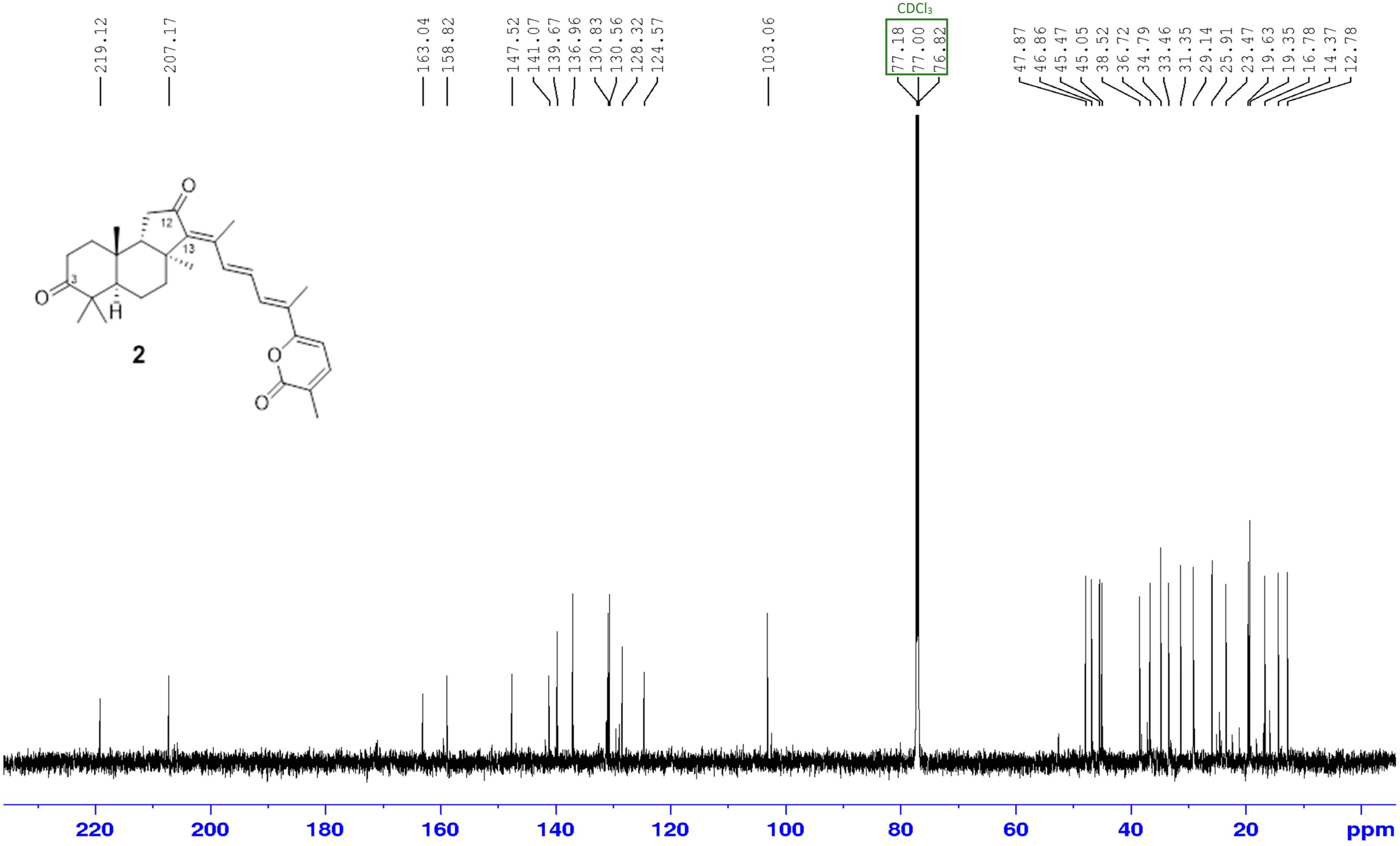

S13 ^{1}H NMR spectrum of stellettin D (**5**) in $CDCl_3$ (700 MHz)

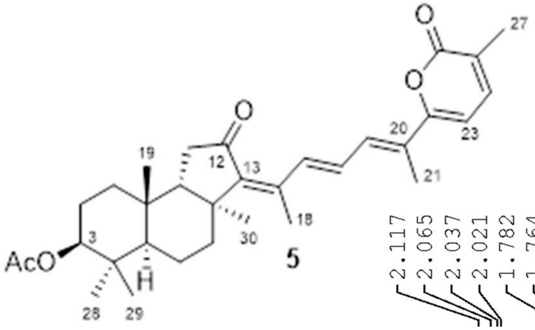

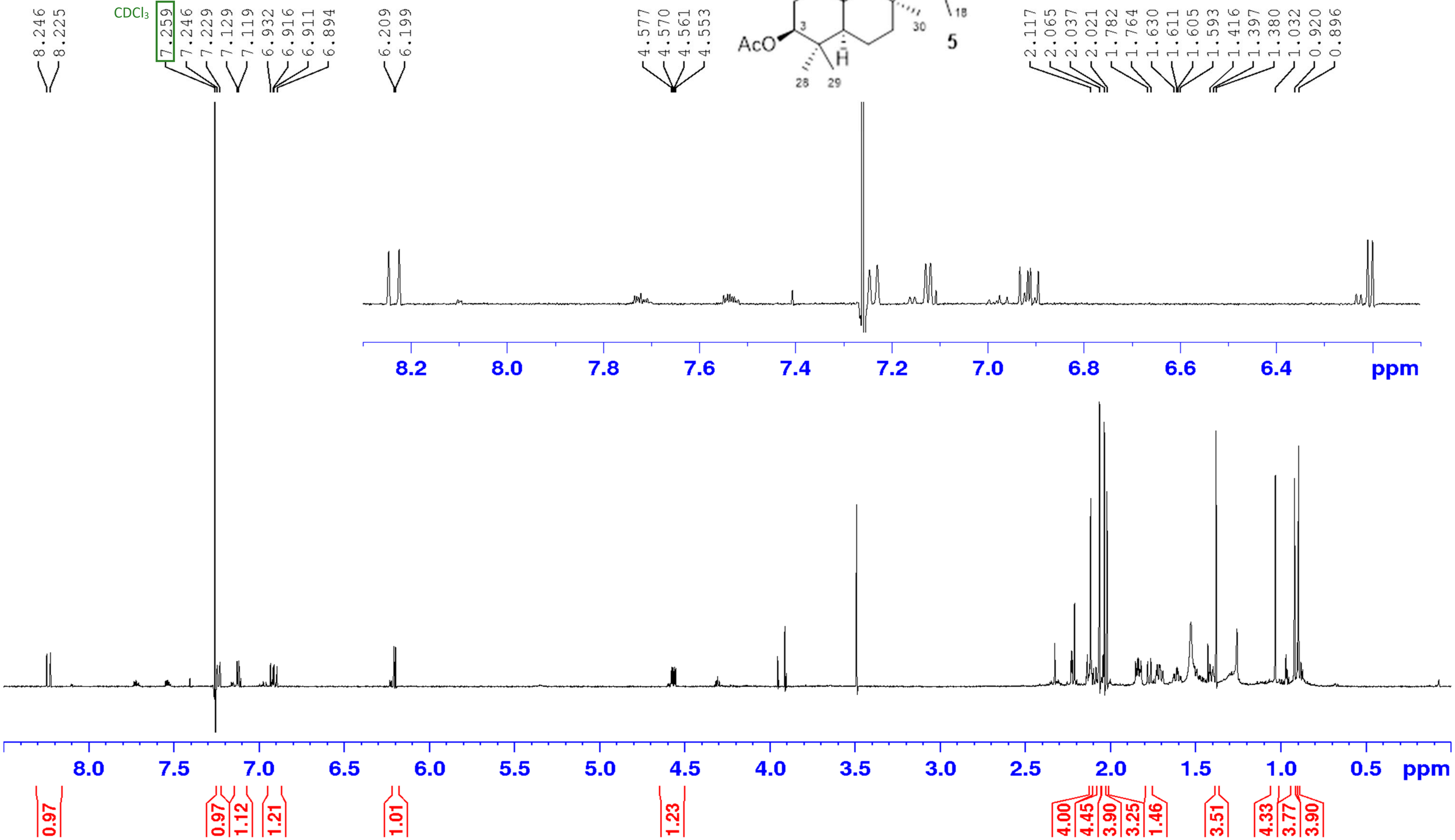

S14 ^{13}C NMR spectrum of stellettin D (**5**) in $CDCl_3$ (176 MHz)

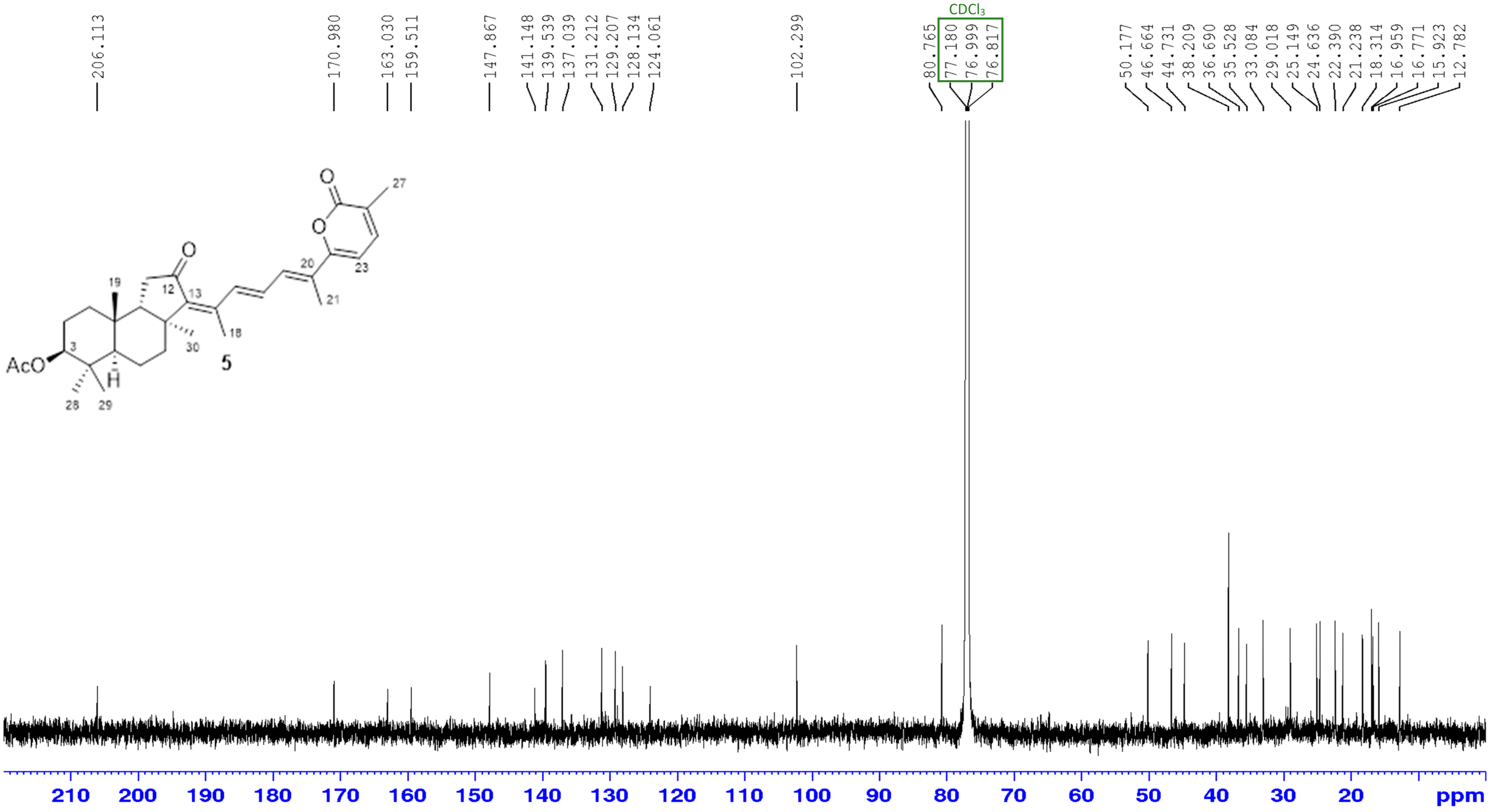

S15 A representative LC–ESI MS chromatograms in negative ion mode for *R. globostellata* samples

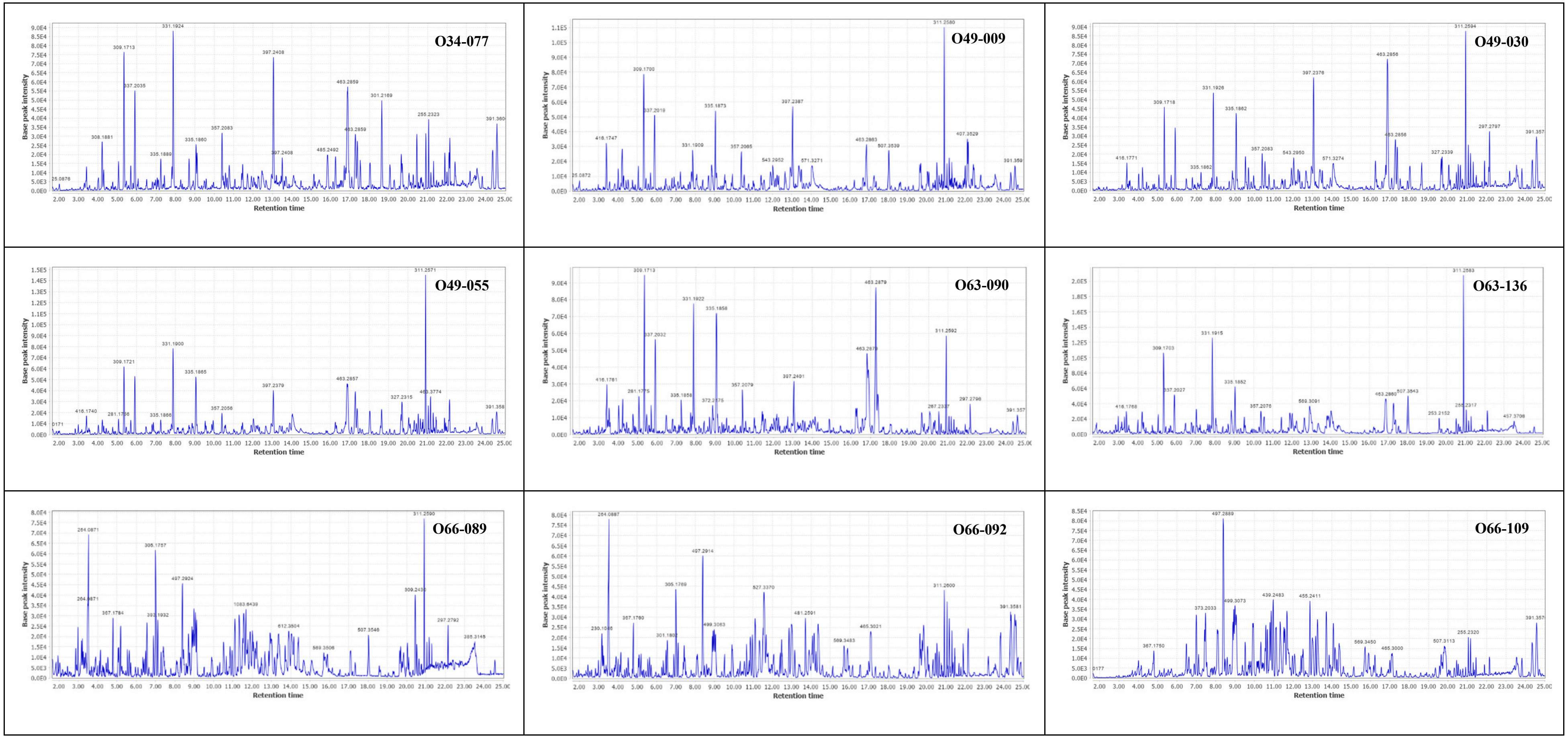

S16 Structures of isomalabaricanes used as standards for LC–ESI MS analyses

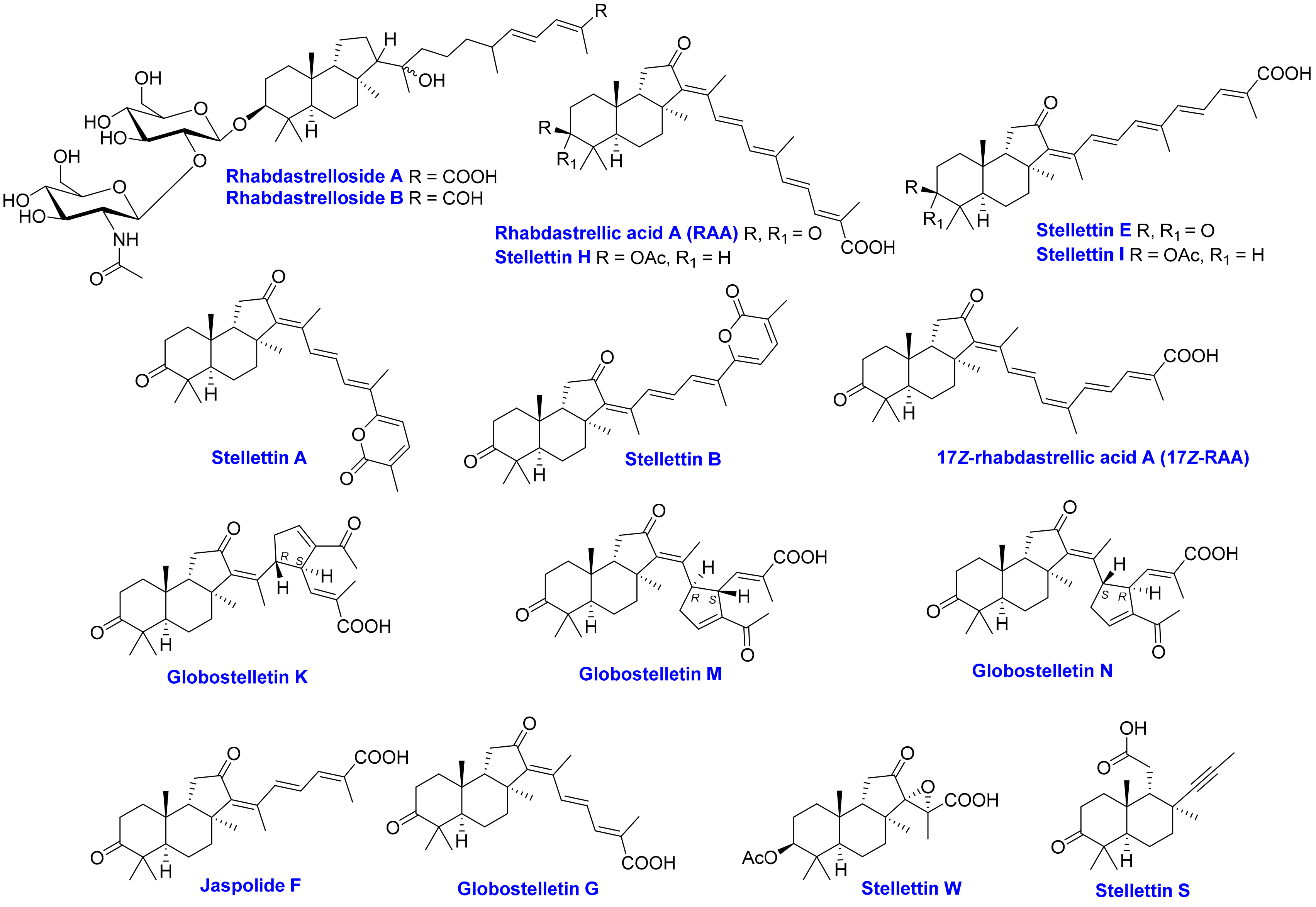

S17 Feature-based molecular network from LC-ESI MS/MS dataset of nine *Rhabdastrella* extracts

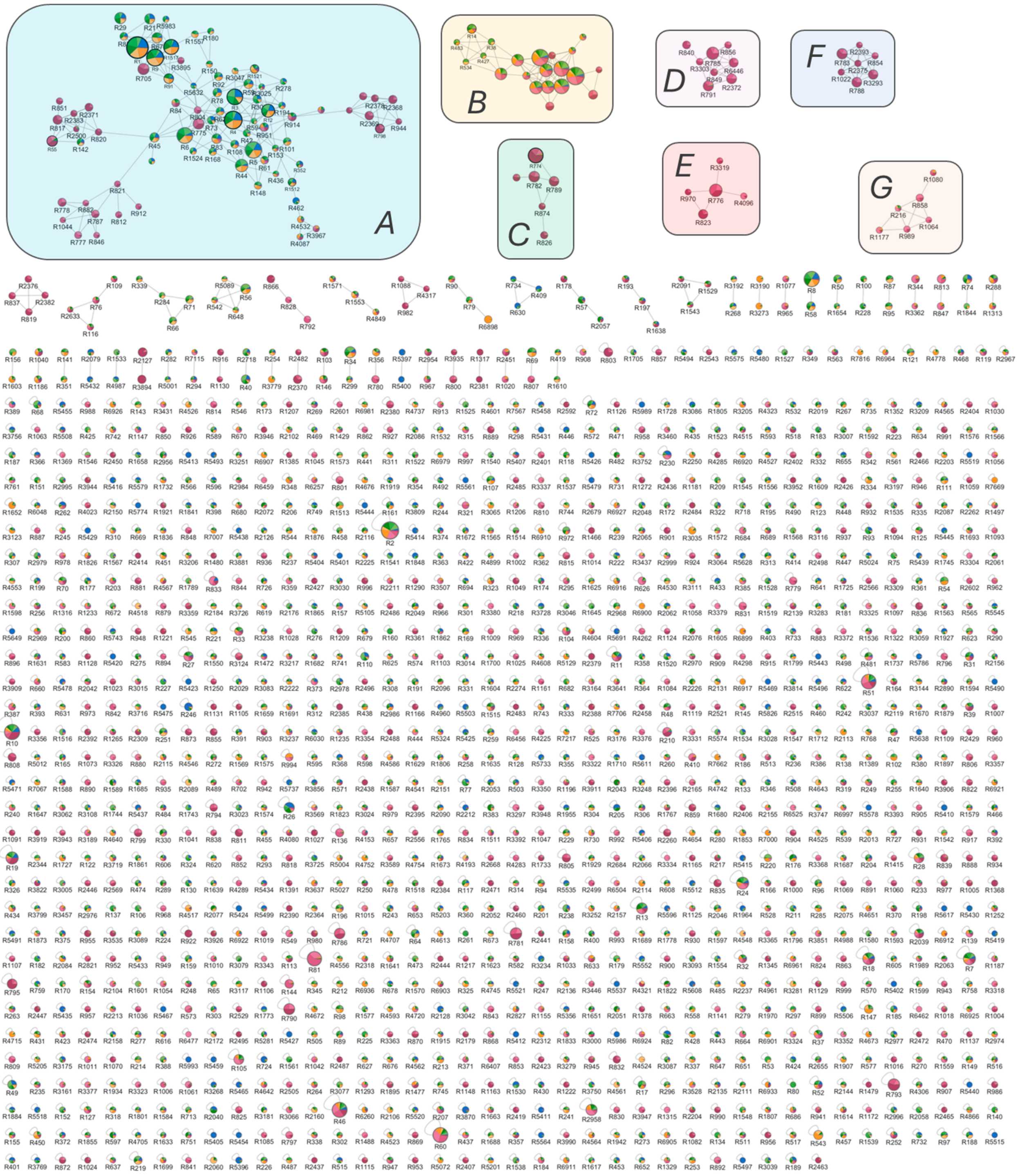

Nodes represent MS/MS features; pie charts indicate the relative contribution of each sample, and edges connect nodes with similar fragmentation spectra.

S18 MS/MS spectra of [M−H]⁻ ions of isomalabaricane standards

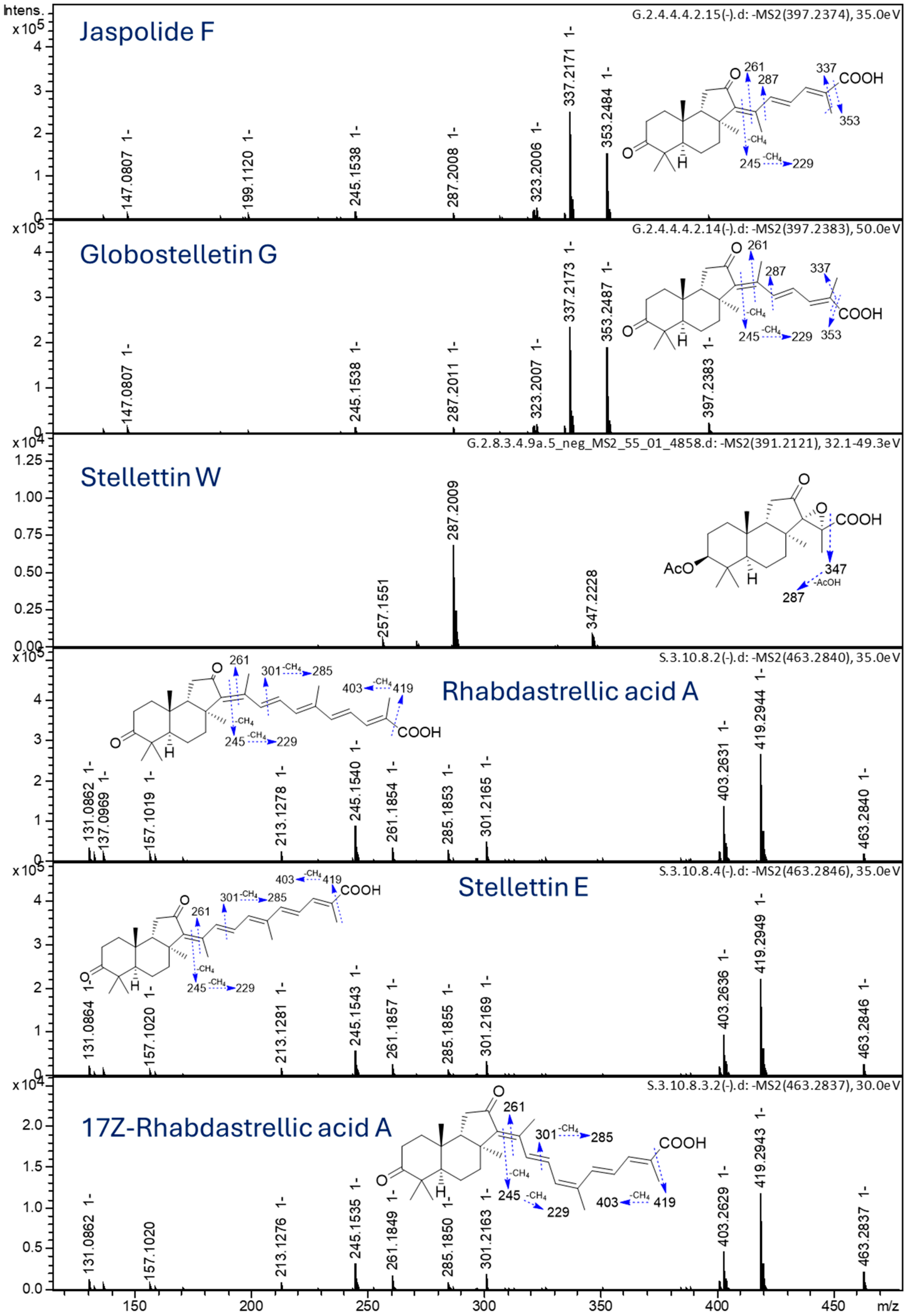

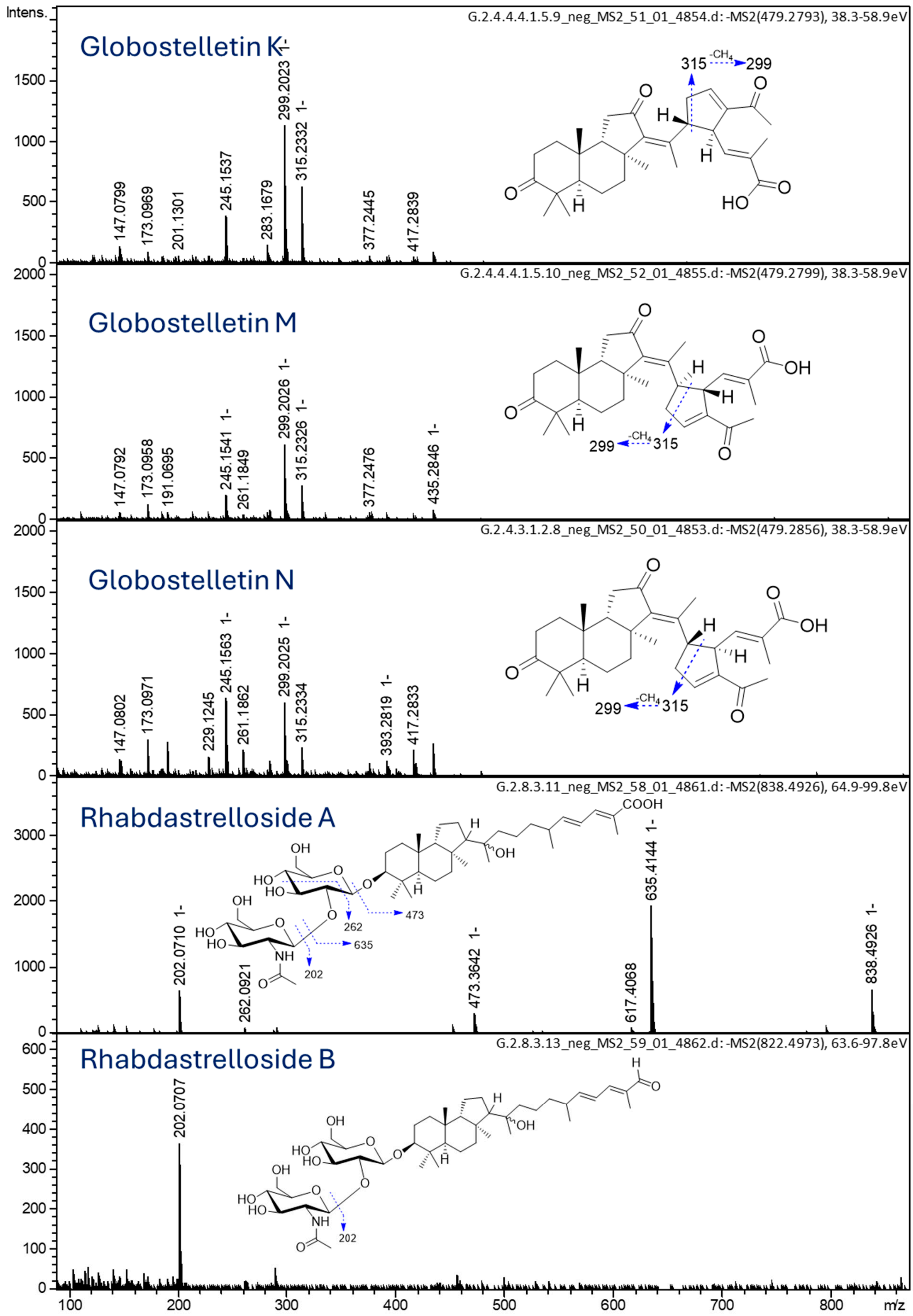
Globostelletin K
G.2.4.4.4.1.5.9_neg_MS2_51_01_4854.d: -MS2(479.2793), 38.3-58.9eV
Globostelletin M
G.2.4.4.4.1.5.10_neg_MS2_52_01_4855.d: -MS2(479.2799), 38.3-58.9eV
Globostelletin N
G.2.4.3.1.2.8_neg_MS2_50_01_4853.d: -MS2(479.2856), 38.3-58.9eV
Rhabdastrelloside A
G.2.8.3.11_neg_MS2_58_01_4861.d: -MS2(838.4926), 64.9-99.8eV
Rhabdastrelloside B
G.2.8.3.13_neg_MS2_59_01_4862.d: -MS2(822.4973), 63.6-97.8eV
Intens.
m/z

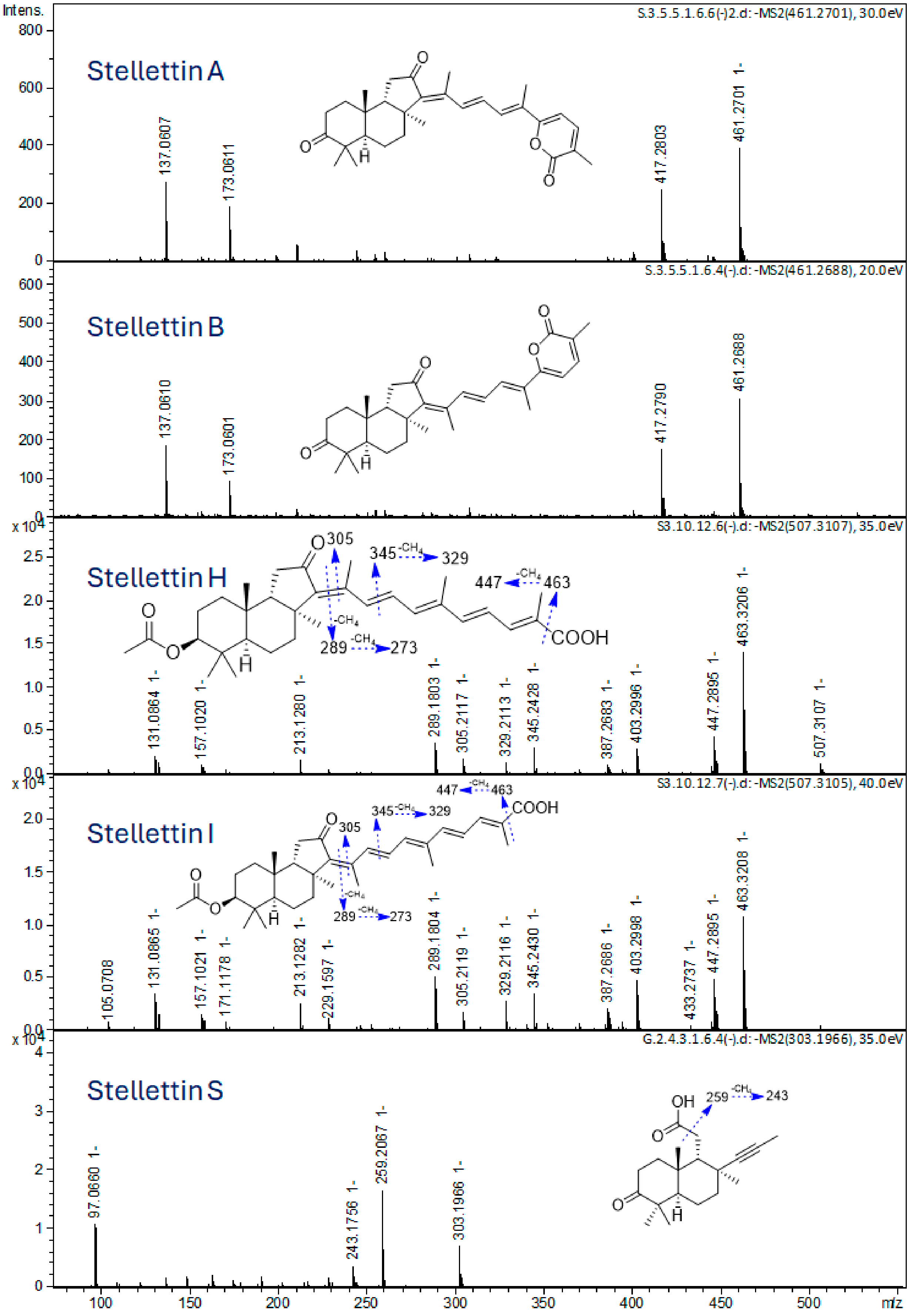

Intens.
S.3.5.5.1.6.6(-)2.d: -MS2(461.2701), 30.0eV
Stellettin A
137.0607
173.0611
417.2803
461.2701 1-
S.3.5.5.1.6.4(-).d: -MS2(461.2688), 20.0eV
Stellettin B
137.0610
173.0601
417.2790
461.2688
S3.10.12.6(-).d: -MS2(507.3107), 35.0eV
Stellettin H
305
345 -CH4 329
447 -CH4 463
289 -CH4 273
COOH
131.0864 1-
157.1020 1-
213.1280 1-
289.1803 1-
305.2117 1-
329.2113 1-
345.2428 1-
387.2683 1-
403.2996 1-
447.2895 1-
463.3206 1-
507.3107 1-
S3.10.12.7(-).d: -MS2(507.3105), 40.0eV
Stellettin I
105.0708
131.0865 1-
157.1021 1-
171.1178 1-
213.1282 1-
229.1597 1-
289.1804 1-
305.2119 1-
329.2116 1-
345.2430 1-
387.2686 1-
403.2998 1-
433.2737 1-
447.2895 1-
463.3208 1-
G.2.4.3.1.6.4(-).d: -MS2(303.1966), 35.0eV
Stellettin S
259 -CH4 243
97.0660 1-
243.1756 1-
259.2067 1-
303.1966 1-
m/z

Table S1. Sizes of spicules of *R. globostellata* (Carter, 1883)

Sample	Oxeas	Orthotriaenes	Spheroxyasters	Oxyasters	Raphides
**PIBOC O63-136**	671.7–**959.6**–1203.0 × 10.7–**17.7**–25.6	rhabds 451.1–**700.6**–892.2 × 12.5–**21.4**–32.5; clades 50.1–**134.3**–210.5 × 9.4–**17.8**–33.5	17.2–**37.5**–64.7	16.7–**27.6**–41.4	68.7–**80.6**–92.4
**PIBOC O66-092**	461.2–**792.0**–1002.5 × 7.5–**13.2**–19.4	rhabds 270.7–**526.9**–822.1 × 5.3–**14.6**–20.5; clades 38.2–**84.1**–122.9 × 5.2–**11.2**–21.6	16.9–**40.7**–59.1	26.4–**48.0**–63.2	not

Table S2. ^{1}H and ^{13}C NMR data of stellettins A (**2**) and D (**5**) in $CDCl_3$

No. [1]	2 (700 and 176 MHz)		5 (700 and 176 MHz)	
	δ_H mult (J in Hz)	δ_C	δ_H mult (J in Hz)	δ_C
1α	2.17, m	31.4	1.61, td (13.2, 4.1)	33.1
1β	1.51, m		1.41, dt (13.2, 3.7)	
2α	2.75, ddd (16.1, 11.9, 5.8)	33.5	1.84, m	25.1
2β	2.38, m		1.72, m	
3α		219.1	4.57, dd (11.7, 5.0)	80.8
4		46.9		38.2
5α	2.44, dd (13.3, 1.8)	45.5	1.77, d (12.3)	46.7
6α	1.67, br dd (13.3, 8.3)	19.6	1.71, m	18.3
6β	1.53, m		1.49, m	
7	2.34, m (Ha-7)	38.5	2.08, m (Hα-7)	38.2
7	2.19, m (Hb-7))		2.12, m (Hβ-7)	
8		45.0		44.7
9β	1.87, t (11.3)	47.9	1.84, m	50.2
10		34.8		35.5
11	2.23 (2H), d (11.3)	36.7	2.22 (2H), m	36.7
12		207.2		206.1
13		147.5		147.9
14		141.1		141.1
15	6.93, d (15.1)	137.0	8.24, d (15.3)	137.0
16	7.01, dd (15.1, 11.3)	130.8	6.91, dd (15.3, 11.5)	131.2
17	7.27, d (11.3)	130.6	7.24, d (11.5)	129.2
18	2.35, s	14.4	2.04, s	15.9
19	0.86, s	23.5	1.03, s	22.4
20		128.3		128.1
21	2.05, s	12.8	2.02, s	12.8
22		158.8		159.5
23	6.24, d (7.0)	103.1	6.20, d (7.0)	102.3
24	7.16, d (7.0)	139.7	7.12, d (7.0)	139.5
25		124.6		124.1
26		163.0		163.0
27	2.14, s	16.8	2.12, s	16.8
28	1.15, s	29.1	0.92, s	29.0
29	1.06, s	19.4	0.90, s	17.0
30	1.45, s	25.9	1.38, s	24.6
<u>C</u>OCH3				171.0
CO<u>CH3</u>			2.06, s	21.2

[1] Assignments were made with the aid of HSQC, HMBC, and ROESY data.

Complete ^{1}H and ^{13}C NMR data for stellettin A (**2**) recorded in $CDCl_3$ have not been reported previously. Also, the ^{1}H NMR signals of stellettin D (**5**) recorded in $CDCl_3$ did not correspond to the previously reported data for the compound [36, see Table S2 in Supplementary materials]. Therefore, we re-investigated the structures of stellettins A and D and performed a complete signal assignment using 2D NMR experiments including COSY, HSQC, HMBC and ROESY.

36. Li, Y. et al. *Fitoterapia*, **2015**, *106*, 226–230. DOI: 10.1016/j.fitote.2015.09.012.

Table S3. Isomalabaricanes in the ethanolic extracts of *R. globostellata* specimens detected by LC–ESI MS/MS and FBMN analysis

ID	RT, min	$[M-H]^-$ meas	Molecular formula	$[M-H]^-$ calc	Error, ppm	Identification
					Cluster A	
R1	16.87	463.2857	$C_{30}H_{40}O_4$	463.2854	-0.7	Rhabdastrellic acid A
R3	13.09	397.2372	$C_{25}H_{34}O_4$	397.2384	3.1	Jaspolide F
R4	7.87	331.1920	$C_{20}H_{28}O_4$	331.1915	-1.6	annotated as Globostelletin B
R5	5.34	309.1711	$C_{17}H_{26}O_5$	309.1707	-1.1	
R6	9.05	335.1872	$C_{19}H_{28}O_5$	335.1864	-2.4	
R9	17.27	463.2858	$C_{30}H_{40}O_4$	463.2854	-0.9	17*Z*-Rhabdastrellic acid A
R12	10.40	357.2075	$C_{22}H_{30}O_4$	357.2071	-1.0	annotated as Globostelletin F
R21	16.24	463.2855	$C_{30}H_{40}O_4$	463.2854	-0.3	annotated as isomer of Rhabdastrellic acid A
R29	16.80	465.2992	$C_{30}H_{42}O_4$	465.3010	3.9	
R30	13.51	397.2389	$C_{25}H_{34}O_4$	397.2384	-1.2	Globostelletin G
R42	12.33	399.2540	$C_{25}H_{36}O_4$	399.2541	0.2	annotated as 15,16-dihydro analogue of Jaspolide F
R44	8.88	372.2171	$C_{22}H_{31}NO_4$	372.2180	2.5	
R45	7.23	335.1865	$C_{19}H_{28}O_5$	335.1864	-0.3	
R55	17.11	465.3007	$C_{30}H_{42}O_4$	465.3010	0.7	annotated as Stellettin K or its isomer
R59	5.06	281.1760	$C_{16}H_{26}O_4$	281.1758	-0.6	
R61	6.79	333.2073	$C_{20}H_{30}O_4$	333.2071	-0.5	
R62	7.85	347.1863	$C_{20}H_{28}O_5$	347.1864	0.3	
R67	16.29	465.3004	$C_{30}H_{42}O_4$	465.3010	1.4	annotated as Stellettin M or L
R73	16.70	441.2646	$C_{27}H_{38}O_5$	441.2646	0.1	
R78	8.70	331.1924	$C_{20}H_{28}O_4$	331.1915	-2.8	annotated as 13*E*-isomer of Globostelletin B
R83	11.46	375.2181	$C_{22}H_{32}O_5$	375.2177	-1.1	annotated as Stellettin X
R84	7.79	337.2026	$C_{19}H_{30}O_5$	337.2020	-1.6	
R85	16.58	463.2853	$C_{30}H_{40}O_4$	463.2854	0.2	annotated as isomer of Rhabdastrellic acid A
R91	20.03	507.3111	$C_{32}H_{44}O_5$	507.3116	1.0	Stellettin H
R92	9.58	399.2178	$C_{24}H_{32}O_5$	399.2177	-0.3	
R101	6.80	390.2276	$C_{22}H_{33}NO_5$	390.2286	2.6	
R108	7.81	353.1974	$C_{19}H_{30}O_6$	353.1970	-1.2	
R142	17.25	465.2986	$C_{30}H_{42}O_4$	465.3010	5.2	annotated as Stellettin K or its isomer
R148	7.64	388.2143	$C_{22}H_{31}NO_5$	388.2129	-3.5	
R150	15.08	504.2780	$C_{31}H_{39}NO_5$	504.2755	-4.9	
R153	9.60	359.2235	$C_{22}H_{32}O_4$	359.2228	-2.0	
R168	7.80	307.1921	$C_{18}H_{28}O_4$	307.1915	-2.0	
R180	14.51	504.2755	$C_{31}H_{39}NO_5$	504.2755	0.1	
R194	10.78	357.2062	$C_{22}H_{30}O_4$	357.2071	2.6	annotated as Globostelletin E
R278	13.25	397.2370	$C_{25}H_{34}O_4$	397.2384	3.6	annotated as isomer of Jaspolide F
R352	12.11	479.2801	$C_{30}H_{40}O_5$	479.2803	0.4	Globostelletin M
R436	5.99	392.2435	$C_{22}H_{35}NO_5$	392.2442	1.9	
R462	13.64	465.2649	$C_{29}H_{38}O_5$	465.2646	-0.5	
R594	12.45	399.2547	$C_{25}H_{36}O_4$	399.2541	-1.5	annotated as 15,16-dihydro analogue of Jaspolide F
R705	19.89	507.3113	$C_{32}H_{44}O_5$	507.3116	0.6	annotated as 22,23-Dihydrostellettin D or its isomer
R775	6.99	305.1772	$C_{18}H_{26}O_4$	305.1758	-4.5	
R777	6.55	405.1937	$C_{22}H_{30}O_7$	405.1919	-4.5	
R778	4.79	367.1769	$C_{19}H_{28}O_7$	367.1762	-1.8	

R787	7.02	409.1870	$C_{21}H_{30}O_8$	409.1868	-0.5	
R798	10.98	439.2493	$C_{27}H_{36}O_5$	439.2490	-0.7	annotated as Jaspiferal A/B or its isomer
R804	6.88	305.1765	$C_{18}H_{26}O_4$	305.1758	-2.2	
R812	6.27	317.1764	$C_{19}H_{26}O_4$	317.1758	-1.8	
R817	9.93	413.2333	$C_{25}H_{34}O_5$	413.2333	0.1	
R820	4.01	295.1554	$C_{16}H_{24}O_5$	295.1551	-1.0	
R821	6.27	421.1877	$C_{22}H_{30}O_8$	421.1868	-2.2	
R846	5.09	383.1717	$C_{19}H_{28}O_8$	383.1711	-1.5	
R851	13.16	395.2230	$C_{25}H_{32}O_4$	395.2228	-0.5	
R882	6.89	409.1874	$C_{21}H_{30}O_8$	409.1868	-1.5	
R912	3.61	379.1754	$C_{20}H_{28}O_7$	379.1762	2.2	
R914	19.70	423.3115	$C_{25}H_{44}O_5$	423.3116	0.2	
R944	10.75	439.2480	$C_{27}H_{36}O_5$	439.2490	2.3	annotated as Jaspiferal A/B or its isomer
R951	17.34	395.2800	$C_{23}H_{40}O_5$	395.2803	0.8	
R1044	5.34	464.2282	$C_{24}H_{35}NO_8$	464.2290	1.7	
R1512	12.28	479.2792	$C_{30}H_{40}O_5$	479.2803	2.3	Globostelletin K
R1517	17.37	463.2852	$C_{30}H_{40}O_4$	463.2854	0.4	Stellettin E
R1521	14.12	423.2521	$C_{27}H_{36}O_4$	423.2541	4.7	annotated as Globostelletin I
R1524	14.18	401.2332	$C_{24}H_{34}O_5$	401.2333	0.4	
R1557	14.28	504.2759	$C_{31}H_{39}NO_5$	504.2755	-0.7	
R2368	14.09	481.2620	$C_{29}H_{38}O_6$	481.2596	-5.1	
R2369	10.86	439.2485	$C_{27}H_{36}O_5$	439.2490	1.1	annotated as Jaspiferal A/B or its isomer
R2371	7.47	373.2030	$C_{22}H_{30}O_5$	373.2020	-2.6	
R2378	13.81	481.2830	$C_{26}H_{42}O_8$	481.2807	-4.8	
R2383	17.00	465.2979	$C_{30}H_{42}O_4$	465.3010	6.7	annotated as Stellettin K or its isomer
R2500	9.78	413.2332	$C_{25}H_{34}O_5$	413.2333	0.4	
R3025	13.16	397.2368	$C_{25}H_{34}O_4$	397.2384	4.1	annotated as isomer of Jaspolide F
R3047	14.25	423.2552	$C_{27}H_{36}O_4$	423.2541	-2.6	annotated as Globostelletin H
R3895	19.75	507.3113	$C_{32}H_{44}O_5$	507.3116	0.6	annotated as 22,23-Dihydrostellettin D or its isomer
R3967	11.66	497.2910	$C_{30}H_{42}O_6$	497.2909	-0.3	
R4087	12.36	495.2765	$C_{30}H_{40}O_6$	495.2752	-2.6	
R4532	11.99	497.2894	$C_{30}H_{42}O_6$	497.2909	2.9	
R5632	8.01	347.1865	$C_{20}H_{28}O_5$	347.1864	-0.3	
R5983	16.38	463.2851	$C_{30}H_{40}O_4$	463.2854	0.6	annotated as isomer of Rhabdastrellic acid A
					Cluster B	
R14	9.53	838.4961	$C_{44}H_{73}NO_{14}$	838.4958	-0.3	Rhabdastrelloside A
R38	9.69	797.4694	$C_{42}H_{70}O_{14}$	797.4693	-0.2	deNAc-analogues of rhabdastrelloside A
R427	9.89	797.4687	$C_{42}H_{70}O_{14}$	797.4693	0.7	deNAc-analogues of rhabdastrelloside A
R483	9.33	838.4962	$C_{44}H_{73}NO_{14}$	838.4958	-0.4	annotated as isomer of Rhabdastrelloside A
R534	9.51	797.4690	$C_{42}H_{70}O_{14}$	797.4693	0.4	deNAc-analogues of rhabdastrelloside A
					Cluster C	
R774	8.41	497.2905	$C_{30}H_{42}O_6$	497.2909	0.7	Globostellatic acid F
R782	11.18	539.3009	$C_{32}H_{44}O_7$	539.3014	1.0	annotated as Globostellatic acid A or its isomer
R789	11.53	539.3004	$C_{32}H_{44}O_7$	539.3014	1.9	annotated as Globostellatic acid A or its isomer
R826	7.49	513.2864	$C_{30}H_{42}O_7$	513.2858	-1.2	
R874	10.24	555.2965	$C_{32}H_{44}O_8$	555.2963	-0.3	
					Cluster D	
R785	9.01	499.3063	$C_{30}H_{44}O_6$	499.3065	0.4	

R791	8.86	497.2905	$C_{30}H_{42}O_6$	497.2909	0.7	
R840	10.32	541.3156	$C_{32}H_{46}O_7$	541.3171	2.7	
R849	8.43	499.3043	$C_{30}H_{44}O_6$	499.3065	4.4	
R856	8.58	499.3060	$C_{30}H_{44}O_6$	499.3065	1.0	
R2372	8.87	499.3024	$C_{30}H_{44}O_6$	499.3065	8.2	
R3303	8.77	499.3043	$C_{30}H_{44}O_6$	499.3065	4.4	
R6446	9.07	499.3061	$C_{30}H_{44}O_6$	499.3065	0.8	
					Cluster E	
R776	11.57	527.3356	$C_{32}H_{48}O_6$	527.3378	4.2	annotated as Globostellatic acid H or I
R823	8.15	499.3050	$C_{30}H_{44}O_6$	499.3065	3.0	
R970	7.93	499.3061	$C_{30}H_{44}O_6$	499.3065	0.8	
R3319	11.36	527.3343	$C_{32}H_{48}O_6$	527.3378	6.7	annotated as Globostellatic acid H or I
R4096	10.51	513.3193	$C_{31}H_{46}O_6$	513.3222	5.6	

Table S4. Primers used for the amplification of 18S and 28S rRNA, and ITS1–5.8S–ITS2 gene fragments

#	Name	Sequence (5’ – 3’)	Region	Temperature anneling (^{0}C)	Authors
1	SP18aF<br>600R18S	CCTGCCAGTAGTCATATGCTT<br>CGAGCTTTTTAACTGCAA		52	Redmond et al. 2007 [65]
2	560F18S<br>1350R18S	GAGGAACAATTGGAGGGC<br>CGGGACTAGTTAGCAGGTTAA		48	Redmond et al. 2007 [65]
3	830F18S<br>1350R18S	TTCGGGACGTTTACTTTG<br>CGGGACTAGTTAGCAGGTTAA		48	Redmond et al. 2007 [65]
4	1200F18S<br>SP18gR	TAATTTGACTCAACACGGG<br>CCTTGTTACGACTTTTACTTCCTC		48	Redmond et al. 2007 [65]
5	1200F18S<br>1800R18S	TAATTTGACTCAACACGGG<br>GTTCACCTACYGAAACCTTGTT		48	Redmond et al. 2007 [65]
6	RA2<br>Demospongiae _28S/ITS_R	GTCCCTGCCCTTTGTACACA<br>GACGTGCCTTTCCAGGTCAACTT		52	Worheide et a1., 2004 [66]
7	Por28S-15F<br>Por28S-878R	GCGAGATCACCYGCTGAAT<br>CACTCCTTGGTCCGTGTTTC	D1-D2	52	Morrow et al, 2012 [67]
8	Por28S-830F<br>Por28S-1520R	CATCCGACCCGTCTTGAA<br>GCTAGTTGATTCGGCAGGTG	D3-D5	52	Morrow et al, 2012 [67]
9	Por28S-1490F<br>Por28S-2170R	AACTCACCTGCCGAATCAAC<br>CCAATCCTTTTCCCAARGTT	D6-D8	52	Morrow et al, 2012 [67]

65. Redmond, N.E. et al. *Mol. Phylogenet. Evol.* **2007**, *43*, 344–352. DOI: 10.1016/j.ympev.2006.10.021
66. Worheide, G. et al. *Mol. Phylogenet. Evol.* **2004**, *33,* 816–830. DOI: 10.1016/j.ympev.2004.07.005
67. Morrow, C.C. et al. *Mol. Phylogenet. Evol.* **2012**, *62*(1), 174–190. DOI: 10.1016/j.ympev.2011.09.016

Table S5. Batch steps and parameters used for data preprocessing in MZmine.

Batch step	Module	Parameters	Value
Raw data import	—	—	—
Mass detection	Wavelet transform	MS1 Noise level	80
		MS2 Noise level	20
		Scale level	5
		Wavelet window size	50%
	Shoulder peaks filter	Peak model function	Lorentzian extended
Chromatogram detection	ADAP Chromatogram Builder	Min group size in number of scans	5
		Group intensity threshold	150
		Min highest intensity	500
		m/z tolerance	0.01 *m/z*
Chromatogram deconvolution	Wavelets (ADAP)	S/N Threshold	8
		Min feature height	500
		Coefficient/area threshold	40
		Peak duration range	0.05–1.0 min
		RT wavelet range	0.0–0.05 min
Isotopic peak removal	Isotopic peaks grouper	*m/z* tolerance	0.02 *m/z*
		Retention time tolerance	0.1 min
Alignment	Join aligner	*m/z* tolerance	0.02 *m/z*
		Weight for *m/z*	50
		Retention time tolerance	0.1 min
		Weight for RT	50
Gap filling	Same RT and m/z range gap filler	*m/z* tolerance	0.01 *m/z*
Filtering	Feature list row filter	Keep only peaks with MS2 scan	
Export results	Export/Submit to GNPS-FBMN with Merge MS/MS	Select spectra to merge	same sample
		m/z merge mode	most intense
		Intensity merge mode	sum intensities
		Expected mass deviation	0.05 *m/z*
		Cosine threshold	70%
		Peak count threshold	20%
		Isolation window offset	0
		Isolation window width	3
